# Novel lures and COI sequences reveal cryptic new species of *Bactrocera* fruit flies in the Solomon Islands (Diptera, Tephritidae, Dacini)

**DOI:** 10.3897/zookeys.1057.68375

**Published:** 2021-08-27

**Authors:** Luc Leblanc, Francis Tsatsia, Camiel Doorenweerd

**Affiliations:** 1 University of Idaho, Department of Entomology, Plant Pathology and Nematology, 875 Perimeter Drive, MS2329, Moscow, Idaho, 83844-2329, USA University of Idaho Moscow United States of America; 2 Biosecurity Solomon Islands. Ministry of Agriculture and Livestock. P.O. Box G13, Honiara, Solomon Islands Ministry of Agriculture and Livestock Honiara Solomon Islands; 3 University of Hawaii, Department of Plant and Environmental Protection Sciences, 3050 Maile Way, Honolulu, Hawaii, 96822-2231, USA University of Hawaii Honolulu United States of America

**Keywords:** *
Dacus
*, Oceania, pest species, taxonomy, *
Zeugodacus
*

## Abstract

Results from a snap-shot survey of Dacine fruit flies carried out on three of the Solomon Islands in April 2018 are reported. Using traps baited with the male lures cue-lure, methyl eugenol, and zingerone, 30 of the 48 species previously known to occur in the Solomon Islands were collected. Six species are newly described here: *Bactroceraallodistincta***sp. nov.**, *B.geminosimulata***sp. nov.**, *B.kolombangarae***sp. nov.**, *B.quasienochra***sp. nov.**, *B.tsatsiai***sp. nov.**, and *B.vargasi***sp. nov.**, all authored by Leblanc & Doorenweerd. An illustrated key to the 54 species now known to be present in the country is provided.

## Introduction

Dacine fruit flies (Diptera: Tephritidae: Dacini), a species-rich Old World tropical group, is composed of 947 currently known species, including 83 crop pests (White and Elson-Harris 1992; Vargas et al. 2015; Doorenweerd et al. 2018). Diversity is particularly high in Australasia, with 332 species described and an imminent publication of 65 new species from Papua New Guinea (R.A.I. Drew, pers. comm.). Many more new species are being discovered, especially cryptic species, with ever improving molecular diagnostic tools and the emergence of new generation male lures (De Meyer et al. 2015; Manrakhan et al. 2017; Royer et al. 2018, 2019; Doorenweerd et al. 2020).

The earliest Dacine fruit fly record in the Solomon Islands was the description of *Bactroceralongicornis* Macquart, in 1835. By 1939, eleven species were known (Malloch 1939), growing to 26 five decades later (Drew 1989). Extensive survey efforts through trapping and host fruit surveys during the Regional Fruit Fly Projects in the Pacific (Allwood and Drew 1997; Allwood 2000; Lidner and McLeod 2008) nearly doubled the number of species to 48 (Drew and Romig 2001). Two decades later, we carried out a snap-shot survey on three islands (Guadalcanal, Kolombangara, Gizo), with the inclusion of zingerone lure, to collect fresh material and develop molecular diagnostic tools to help further characterize the species found in the Solomon Islands. In just a couple of weeks, we discovered six new species, including cryptic species that would not have been detectable without molecular characterization. We herein describe these new species and provide a key to the 54 species now present in the Solomon Islands.

## Materials and methods

### Collecting and curation

**Figure 1.**
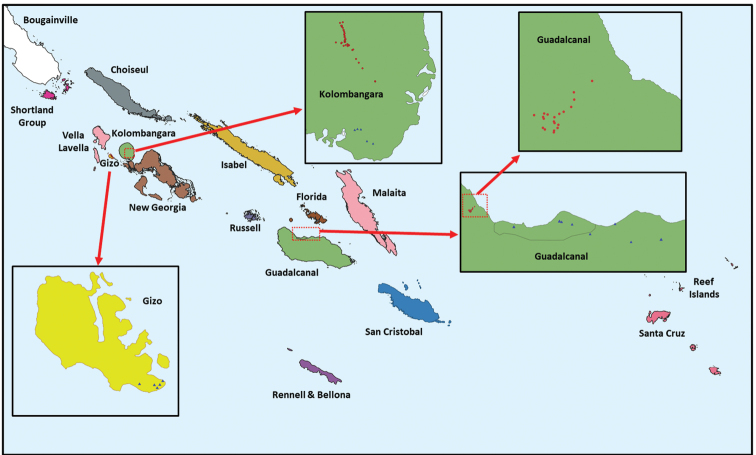
Map of the Solomon Islands, with groups of islands for distribution records, and trapping locations on Guadalcanal, Gizo and Kolombangara islands in the 2018 survey. Red circles are sites located in forest and blue triangles are sites in agricultural environments.

We maintained 79 sets of three traps separately baited with male lures (cue-lure, methyl eugenol and zingerone) in the Solomon Islands in April 2018. We used commercially available cue-lure and methyl eugenol plugs (Scentry Biologicals, Billings, Montana). Zingerone (= vanillylacetone) lure was prepared by dipping dental cotton wicks in zingerone powder (Sigma-Aldrich) melted over a hot plate and allowed to solidify in the wicks. Small vertical bucket traps (Leblanc et al. 2015: fig. 1) were made of 5-oz urine sample cups (Stockwell Scientific, Scottsdale, Arizona) with two 20 mm wide lateral circular openings on opposite sides, 12 mm below the top, with a hole drilled in the lid center, through which a 30-cm-long, 15-gauge, aluminum tie wire was inserted, and bent into a hook below the lid. The male lure unit and a 10 × 10 mm piece of dichlorvos (DVVP) strip (Vaportape II; Hercon Environmental, Emingsville, PA) were attached to the hook below the lid. A 10-cm-wide black square plastic food plate (Waddington North America) was placed on top of the trap to prevent flooding by frequent rain. A solution of 25% propylene glycol (Better World Manufacturing, Fresno, CA) was used in the trap to preserve captured flies, until they were transferred to 95% ethanol during trap servicing. The 79 sets of traps in agricultural areas and endemic forest on the islands of Guadalcanal and Kolombangara, and agricultural areas on Gizo Island (Fig. 1) were maintained for 12, four, and six days, respectively. Forest trapping sites were ca. 50 meters apart along transects that followed trails. Sampled flies were stored in 95% ethanol in a -20 °C freezer to preserve DNA. All flies were identified to species using available keys (Drew 1989; Drew and Romig 2001). We pulled one or two legs from specimens that were selected for DNA extraction (for further details on DNA extraction methods see Doorenweerd et al. 2020). All holotypes and all, or a subset of, the paratypes were double-mounted to be stored as dry specimens in collections for permanent future reference. Before drying flies for double-mounting (White and Elson-Harris 1992), we pinned them through the scutum with a minuten pin and soaked them in diethyl-ether for 3–12 hours to fix and preserve their natural coloration. We photographed specimens using a Nikon D7100 camera attached to an Olympus SZX10 microscope and used Helicon Focus pro v6.7.1 to merge pictures taken at a range of focal planes. To measure specimens (all available or up to 10 specimens measured per species), we used an ocular grid mounted on an Olympus SZ30 dissecting microscope.

### Morphological terms and taxonomic assignment

Morphological terminology used in the descriptions follows White et al. (1999) and assignment of species to genera follows Doorenweerd et al. (2018). We treat *Zeugodacus* as a distinct genus from *Bactrocera* and *Dacus* (Krosch et al. 2012; Virgilio et al. 2015; Dupuis et al. 2017; San Jose et al. 2018). Subgenus assignment for each species follows reclassifications recently published by Hancock and Drew (Hancock 2015; Drew and Hancock 2016; Hancock and Drew 2015, 2018a, b). The host plant records included in the key follow the compilation published by Leblanc et al. (2012). For accurate taxonomic application of host plant records from the literature we used the World Flora Online (WFO 2021).

**Figure 2.**
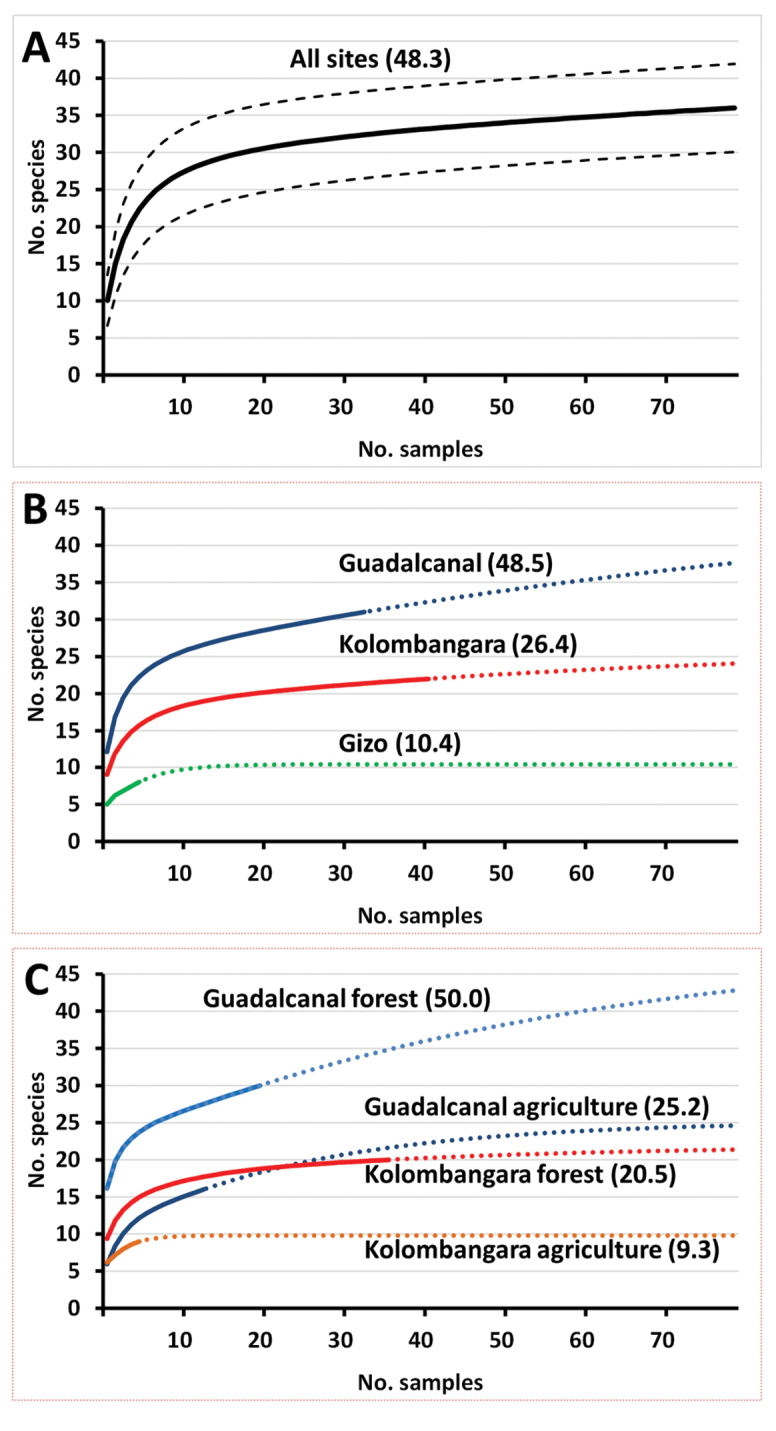
Species accumulation curves based on the 2018 survey of Solomon Islands **A** for all sampled sites with 95% confidence interval range **B** for the three individually sampled islands, and **C** comparing forest and agricultural sites separately on Guadalcanal and Kolombangara. Estimated species numbers for each curve (in brackets) based on the Chao 2 estimator.

### COI sequence analysis

Representatives of all species newly described here were also included in the cytochrome c oxidase I (COI) study of Doorenweerd et al. (2020), under tentative species names. For that study, 1493 base pairs of the COI gene were sequenced and comparatively analyzed in a dataset that included 163 species of *Bactrocera*. We include here the maximum likelihood gene tree from that study and the summary *Bactrocera* species statistics as supplementary material (Suppl. material 1: Fig. S1, Suppl. material 2: Table S1). For the methods for DNA extraction, sequencing and analyses we refer to Doorenweerd et al. (2020). Collecting information as well as COI sequences are available on BOLD (Ratnasingham and Hebert 2007) dataset (DOI: http://dx.doi.org/10.5883/DS-DACCOI), as well as NCBI GenBank (accessions MZ196488–MZ196507). Each specimen for which DNA was extracted was assigned a unique code in the format “UHIM.ms00000”, physically labelled as such, and this number forms the ‘Sample ID’ in BOLD.

### Estimating biodiversity

We used EstimateS software (Colwell 2019) to generate species accumulation curves and estimate species diversity, using the incidence-based Chao 2 algorithm. We generated accumulation curves, with 100 randomizations without replacement for confidence intervals for all sites collectively, separately for each island, and comparing agricultural and forest sites in Guadalcanal and Kolombangara.

### Abbreviations

**BPBM**Bernice Pauahi Bishop Museum, Honolulu, Hawaii, United States;

**BSI** Biosecurity Solomon Islands, Honiara, Solomon Islands;

**WFBM** William F. Barr Entomological Museum, Moscow, Idaho, United States;

**UHIM** University of Hawaii Insect Museum, Honolulu, Hawaii, United States;

**USNM**National Museum of Natural History, Smithsonian Institution, Washington DC, United States.

## Results and taxonomy

### Bactrocera (Bactrocera) allodistincta

Taxon classificationAnimaliaDipteraTephritidae

Leblanc & Doorenweerd sp. nov.

931B15D8-77B4-5D1C-91A7-9A2DADE1B808

http://zoobank.org/6D929FD2-D802-42D1-B15D-B14C78CF4442

Fig. 3A–E

#### Type material.

***Holotype*.** Solomon Islands • ♂; Guadalcanal, forest; -9.4067, 159.8647; 167 m; 4–16 Apr. 2018; L. Leblanc, F. Tsatsia leg.; cue-lure baited trap FFSo015. Deposited in UHIM. ***Paratypes*.** 11 males. Solomon Islands • 1 ♂; Guadalcanal forest; -9.4041, 159.8628; 153 m; 4–16 Apr. 2018; L. Leblanc, F. Tsatsia leg.; cue-lure baited trap FFSo011 • 1 ♂; same locality and date as for preceding; -9.4067, 159.8647; 167 m; trap FFSo015 • 1 ♂; same locality and date as for preceding; -9.4072, 159.8664; 153 m; trap FFSo016 • 2 ♂; same locality and date as for preceding; -9.4064, 159.8671; 145 m; trap FFSo018; molecular voucher UHIM.ms08766 • 2 ♂; same locality and date as for preceding; -9.4059, 159.8672; 133 m; trap FFSo019 • 1 ♂; same locality and date as for preceding; -9.4055, 159.8665; 145 m; trap FFSo020 • 1 ♂; same locality and date as for preceding; -9.4040, 159.8652; 125 m; trap FFSo023 • 1 ♂; same locality and date as for preceding; -9.4026, 159.8695; 57 m; trap FFSo027 • 1 ♂; same locality and date as for preceding; -9.4000, 159.8700; 57 m; trap FFSo029. Seven of the paratypes are deposited at UHIM, three at WFBM, and one at USNM.

#### Differential diagnosis.

**Figure 3.**
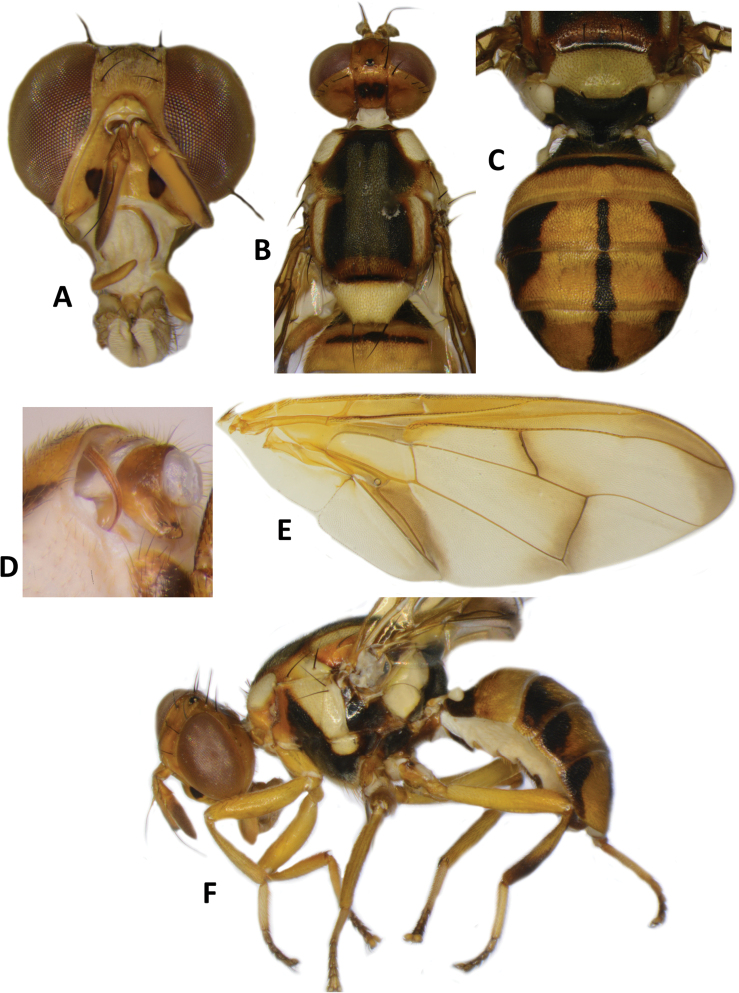
*Bactroceraallodistincta* sp. nov. **A** head **B** head and scutum **C** abdomen **D** male genitalia **E** wing **F** lateral view.

**Figure 4.**
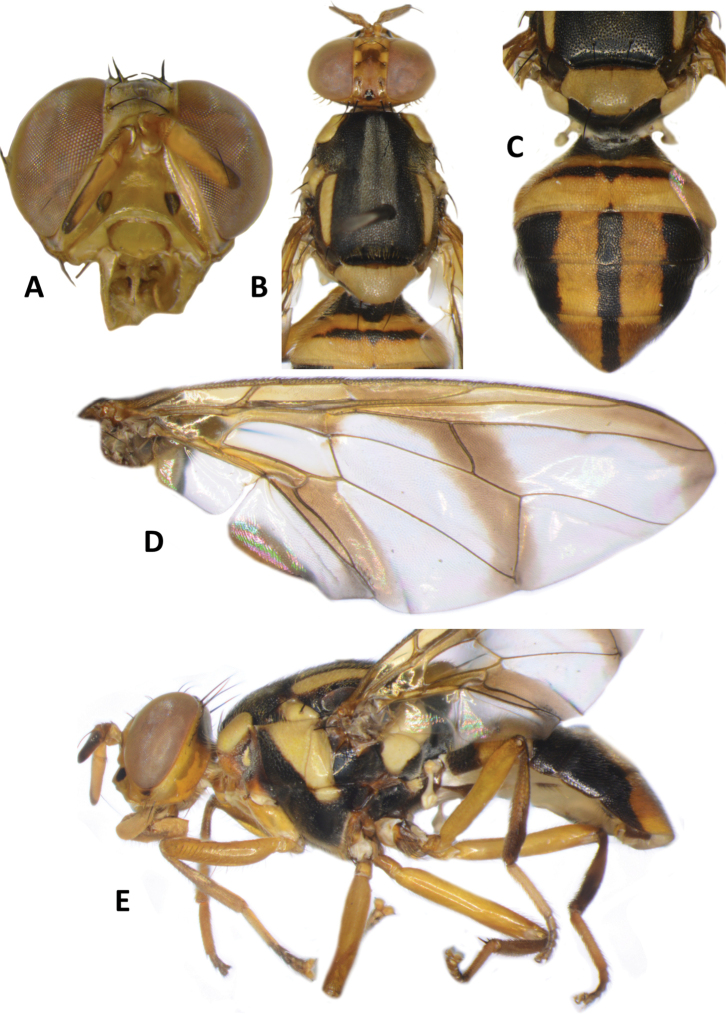
*Bactrocerapseudodistincta* (Drew) **A** head **B** head and scutum **C** abdomen **D** wing **E** lateral view.

**Figure 5.**
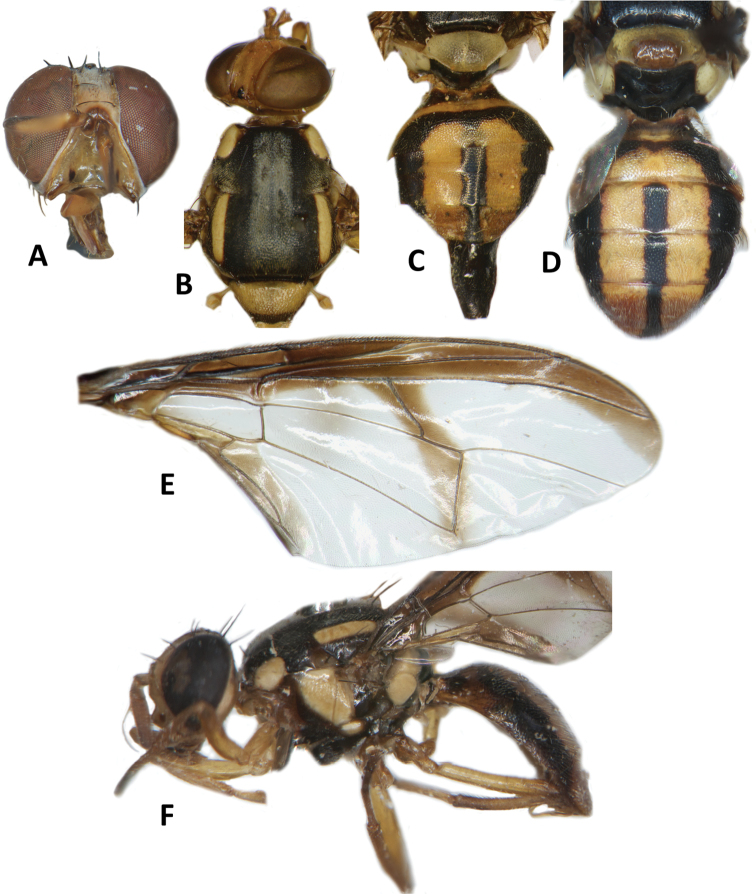
*Bactroceradistincta* (Malloch) **A** head **B** head and scutum **C** female abdomen **D** male abdomen **E** wing **F** lateral view.

*Bactroceraallodistincta* differs from *B.pseudodistincta* (Drew) (Fig. 4) in the presence of orange-brown lateral and posterior markings on the predominantly black scutum, abdominal tergites III–V with a narrower medial black stripe, the lateral black markings on tergite IV narrowed posteriorly, and the rather diffuse fuscous crossband on the wing. It differs from *B.distincta* (Malloch) in that the costal band is diffuse orange-brown and the crossband is sinuous, with a bend along vein M (Fig. 3E), whereas the entire costal band, including in the basicostal and costal cells, is dark fuscous and the crossband is broad and straight in *B.distincta* (Fig. 5E).

#### Molecular diagnosis.

We obtained a single COI sequence (UHIM.ms08766) which matches closest to *Bactrocerapedestris* (Bezzi) [misidentified as *B.gombokensis* Drew & Hancock, 1994 in Doorenweerd et al. 2020], at 10.25% pairwise distance. *Bactrocerapseudodistincta* (Drew) [N = 2] is also represented in the dataset and does not appear as a close match, but *B.distincta* is not represented.

#### Description of adult.

**Male. *Head*** (Fig. 3A). Height 1.56 ± 0.12 (SD) (1.37–1.67) mm. Frons of even width, 0.80 ± 0.03 (0.73–0.83) mm long and 1.40 ± 1.05 (1.33–1.50) times as long as broad; fulvous, sometimes fuscous around orbital seta and anteromedial hump; latter covered by short red-brown microtrichia; three pairs of dark fuscous frontal setae present; lunule fulvous. Ocellar triangle black. Vertex fulvous with two pairs of dark fuscous vertical setae. Face fulvous with a pair of moderately sized oval black spots in antennal furrows; length 0.49 ± 0.05 (0.43–0.60) mm. Gena fulvous, with a fuscous subocular spot and a dark fuscous seta. Occiput fulvous with a dark fuscous to black dorsomedial marking; a row of 4–6 dark fuscous postocular setae present behind eye. Antenna with scape and pedicel fulvous and first flagellomere fulvous with pale fuscous on lateral surface of flagellum; a strong red-brown dorsal seta on pedicel; arista fulvous basally and black distally; length of segments: 0.22 ± 0.04 (0.17–0.27) mm; 0.27 ± 0.03 (0.23–0.33) mm; 0.71 ± 0.04 (0.67–0.73) mm.

***Thorax*** (Fig. 3B). Scutum black except orange-brown ventral to and narrowly medial to lateral postsutural vitta, around notopleural suture, along lateral margin between postpronotal lobe and notopleuron, medial to postpronotal lobe, and along posterior margin of scutum. Scutum with two broad parallel medial stripes of dense silvery microtrichia along entire scutum length. Pleural areas black except orange-brown anterior margin of anepisternum and proeipsternum. Yellow markings: postpronotal lobe; notopleuron; moderately broad paired parallel-sided lateral postsutural vitta reaching intra-alar seta posteriorly; broad anepisternal stripe with anterior margin straight, reaching to anterior notopleural seta dorsally; a large transverse spot on katepisternum below the anepisternal stripe; anterior 4⁄5 of anatergite and ¾ of katatergite (posteriorly black). Mediotergite black. Scutellum yellow except for very narrow black basal band. Setae: 1 pair scutellar; 1 pair prescutellar acrostichal; 1 pair intra-alar; 1 pair postalar; 1 pair postsutural supra-alar; 1 pair anepisternal; 2 pairs notopleural; 2 pairs scapular; all setae well developed and dark fuscous.

***Legs*** (Fig. 3F). All legs entirely fulvous with apical 2⁄5 of hind tibia fuscous. Fore femur with a row of long pale dorsal setae. Mid-tibia with apical black spur.

***Wing*** (Fig. 3E). Length 5.6 ± 0.2 (5.3–5.9) mm; basal costal and costal cells fuscous with microtrichia in posterodistal corner of costal cell; broad fuscous costal band confluent with R_4+5_, remaining broad at apex and ending at apex of medial vein; a diffuse orange-brown crossband along crossvein r-m, continuing along M and dm-cu to reach posterior wing margin, and a broad fuscous anal streak over cell bcu and basal margin of cu_1_; remainder of wing light fuscous; dense aggregation of microtrichia around A_1_ + CuA_2_; supernumerary lobe weakly developed.

***Abdomen*** (Fig. 3C, D). Oval with tergites not fused; pecten present on tergite III; posterior lobe of surstylus short; abdominal sternite V with a deep concavity on posterior margin. Base of syntergite I+II wider than long. Syntergite I+II orange-brown with base black and a narrow sub-basal transverse medial black band. Tergites III–V orange-brown with moderately broad medial black stripe reaching apex, and large lateral black markings on tergite III and anterolateral corners of tergites IV and V. Ceromata on tergite V indistinct from abdomen orange-brown color. Sternite I dark fuscous, sternite II fulvous, and sternites III–V fulvous tending fuscous medially.

**Female.** Unknown

#### Male attractant.

Cue-lure.

#### Etymology.

The specific name is a noun in apposition, derived from the Greek *allos* (another) and the species resembles *B.distincta* (Malloch). Previously, *B.pseudodistincta* (Drew) had been described as a species with similar appearance to *B.distincta*. All three are present in Oceania.

#### Notes.

*Bactroceraallodistincta* was included as *B.* spnSol01 in Doorenweerd et al. (2020).

### Bactrocera (Bactrocera) geminosimulata

Taxon classificationAnimaliaDipteraTephritidae

Leblanc & Doorenweerd sp. nov.

E79030C5-5986-531F-96D6-4897A8AD7931

http://zoobank.org/49835D53-30EE-47F9-9F64-DE320C2E046A

Fig. 6A–E , 9E–G

#### Type material.

***Holotype*.** Solomon Islands • ♂; Guadalcanal, forest; -9.4045, 159.8665; 120 m; 4–16 Apr. 2018; L. Leblanc, F. Tsatsia leg.; cue-lure baited trap FFSo022; molecular voucher UHIM.ms09156”. Deposited in UHIM. ***Paratypes*.** 13 males. Solomon Islands • 4 ♂; Guadalcanal, forest; -9.4072, 159.8664; 153 m; 4–16 Apr. 2018; L. Leblanc, F. Tsatsia leg.; cue-lure baited trap FFSo016; molecular voucher UHIM.ms08673 • 2 ♂; same locality and date as for preceding; -9.4069, 159.8664; 153 m; trap FFSo017 • 2 ♂; same locality and date as for preceding; -9.4064, 159.8671; 145 m; trap FFSo018 • 1 ♂; same locality and date as for preceding; -9.4045, 159.8665; 139 m; trap FFSo022 • 2 ♂; same locality and date as for preceding; -9.4038, 159.8646; 103 m; trap FFSo024; molecular voucher UHIM.ms09155) • 2 ♂; same locality and date as for preceding; -9.4026, 159.8695; 57 m; trap FFSo027; molecular vouchers UHIM.ms09153, UHIM.ms09154. Nine of the paratypes are deposited at UHIM, three at WFBM, and one at USNM.

#### Differential diagnosis.

*Bactrocerageminosimulata* is identical in all points to the sympatric *B.simulata* (Malloch), only distinguished by a subtle difference in wing infuscation in the presence of a light fuscous tinge as a broad, somewhat triangular area covering much of the middle of the wing, including the areas bordering r-m and dm-cu (Fig. 9E–G); the latter is absent in *B.simulata* (Fig. 9A–D). The new species can be distinguished from *B.bryoniae* (Tryon) by the lighter fuscous tinge of the costal band, a narrower anal streak and the largely to entirely black abdomen, whereas the abdomen in *B.bryoniae* is orange-brown with a narrow black ‘T’-shaped pattern (Fig. 8). *Bactrocerabryoniae* is widespread in Australia and New Guinea but is absent from the Solomon Islands.

#### Molecular diagnosis.

The COI sequences of *B.geminosimulata* [N = 4] are similar to those of *B.bryoniae* [N = 5], but with a minimum of 1.47% pairwise distance. The reference COI dataset only includes *B.bryoniae* from Australia. The COI sequences suggest no close relationship with *B.simulata*, and can be used to reliably distinguish *B.geminosimulata* from *B.simulata*.

#### Description of adult.

**Figure 6.**
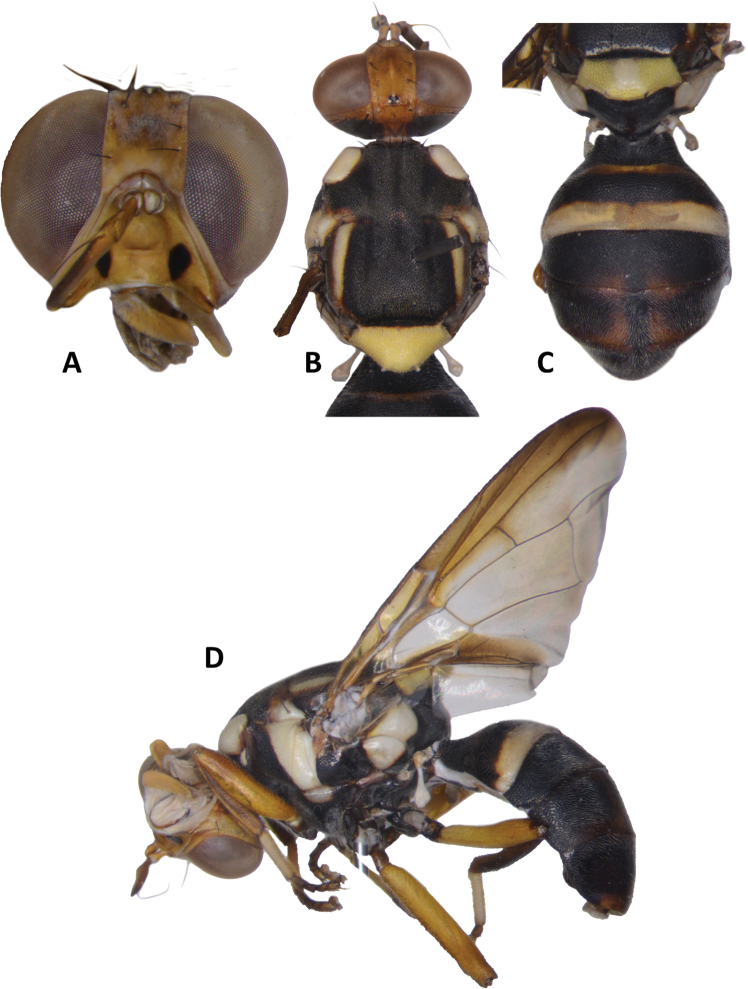
*Bactrocerageminosimulata* sp. nov. **A** head **B** head and scutum **C** abdomen **D** lateral view and wing.

**Male. *Head*** (Fig. 6A). Height 2.02 ± 0.18 (SD) (1.77–2.17) mm. Frons, of even width, 0.98 ± 0.11 (0.83–1.07) mm long and 1.33 ± 0.08 (1.24–1.43) times as long as broad; generally fulvous; anteromedial hump covered by short red-brown microtrichia; three pairs of black frontal setae present; lunule yellow. Ocellar triangle black. Vertex fulvous with two pairs of black vertical setae. Face fulvous with a pair of large circular black spots in antennal furrows; length 0.62 ± 0.07 (0.53–0.67) mm. Gena fulvous, with or without a faint dark fuscous subocular spot; red-brown seta present. Occiput dark fuscous and narrowly fulvous along eye margin; a row of 6–8 black postocular setae present behind eye. Antenna with scape and pedicel fulvous and flagellum fulvous with light fuscous lateral surface; a strong red-brown dorsal seta on pedicel; arista fulvous basally and black distally; length of segments: 0.30 ± 0.03 (0.27–0.33) mm; 0.40 ± 0.05 (0.33–0.43) mm; 0.95 ± 0.07 (0.89–1.03) mm.

**Figure 7.**
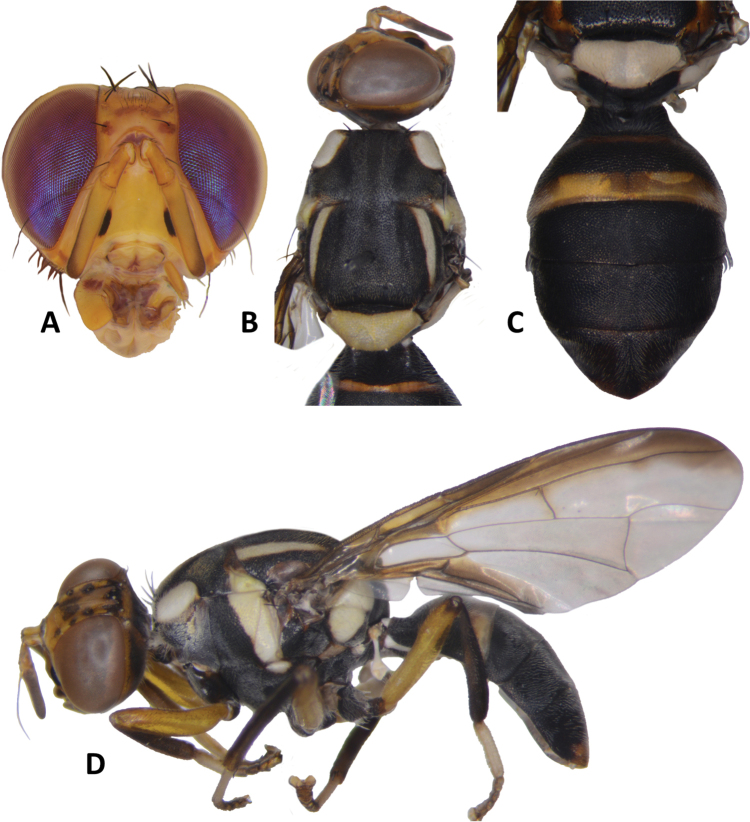
*Bactrocerasimulata* (Malloch) **A** head **B** head and scutum **C** abdomen **D** lateral view and wing.

***Thorax*** (Fig. 6B). Scutum black with small orange-brown markings anterior and posterior to lateral postsutural vitta. Pleural areas black. Yellow markings: postpronotal lobe; notopleuron; moderately broad paired lateral postsutural vitta, tapering posteriorly and ending before intra-alar seta posteriorly; moderately broad anepisternal stripe with anterior margin straight, ending before anterior notopleural seta dorsally; a large transverse spot on katepisternum below the anepisternal stripe; anterior ¾ of anatergite and katatergite (posteriorly black). Mediotergite black. Scutellum yellow with narrow black basal band. Setae: 1 pair scutellar; 1 pair prescutellar acrostichal; 1 pair intra-alar; 1 pair postalar; 1 pair postsutural supra-alar; 1 pair anepisternal; 2 pairs notopleural; 2 pairs scapular; all setae well developed and black.

***Legs*** (Fig. 6E). Coxae and trochanters black. Remainder of legs fulvous with hind tibia tending fuscous to dark fuscous. Fore femur with a row of long dark dorsal setae. Mid-tibia with an apical black spur.

***Wing*** (Fig. 9E–G). Length 6.4 ± 0.4 (5.9–6.9) mm; basal costal and costal cells fulvous with microtrichia in posterodistal corner of costal cell; broad dark fuscous costal band confluent with R_4+5_, ending between R_4+5_ and medial vein; light fuscous tinge as a broad, somewhat triangular area covering much of the middle of the wing, including the areas bordering r-m and dm-cu (absent in *B.simulata*); broad dark fuscous anal streak; dense aggregation of microtrichia around A_1_ + CuA_2_; supernumerary lobe moderately developed.

***Abdomen*** (Fig. 6C, D). Oval with tergites not fused; pecten present on tergite III; posterior lobe of surstylus short; abdominal sternite V with a deep concavity on posterior margin. Base of syntergite I+II wider than long. Syntergite I+II black except for yellow along posterior half of and narrowly orange-brown along anterior margin of tergite II. Tergites III–V entirely black or with two broad longitudinal orange-brown areas running from center of tergite IV to posterior margin of tergite V, each side of a broad medial longitudinal dull black stripe. Ceromata on tergite V black. Abdominal sternites black.

**Female.** Unknown

#### Male attractant.

Cue-lure.

#### Etymology.

The specific name is a noun in apposition, derived from the Latin noun *geminus* (twins) and the epithet of the sympatric and morphologically nearly identical *B.simulata* (Malloch).

**Figure 8.**
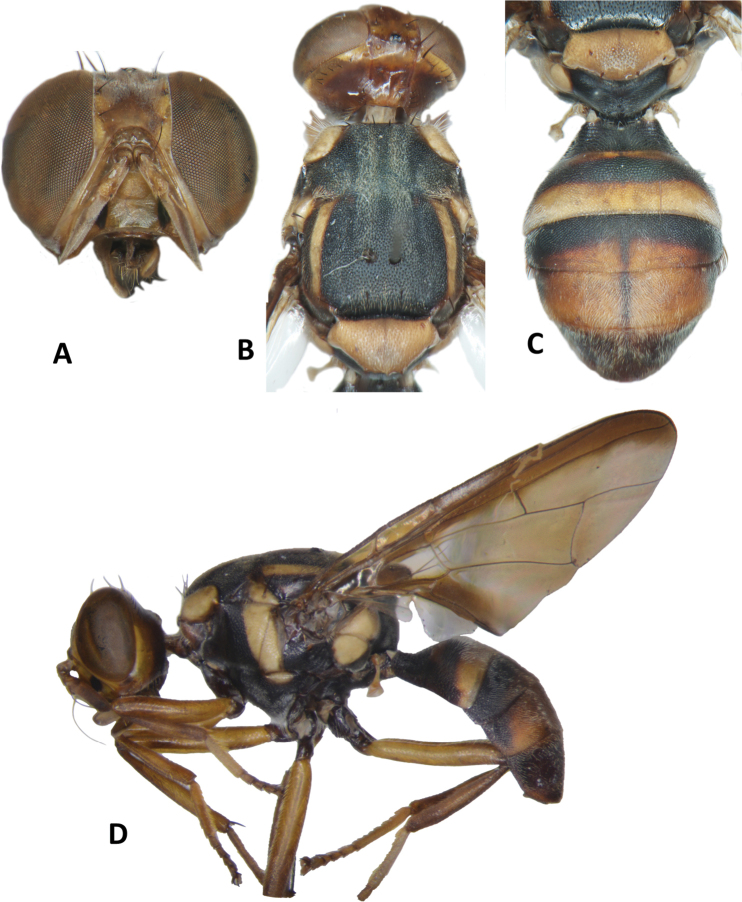
*Bactrocerabryoniae* (Tryon) **A** head **B** head and scutum **C** abdomen **D** lateral view and wing.

#### Notes.

*Bactrocerageminosimulata* was included as *B.* spSol12 in Doorenweerd et al. (2020).

**Figure 9.**
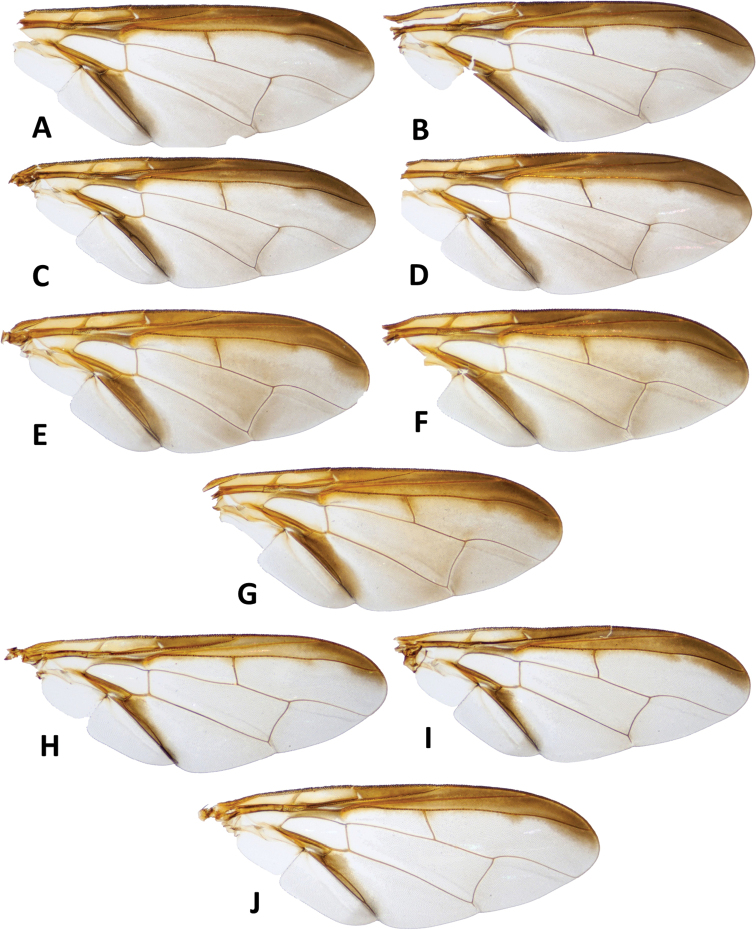
Wings of *Bactrocerasimulata* (Malloch) **A** molecular voucher ms09146 **B** ms09147 **C** ms09148 **D** ms09151, *Bactrocerageminosimulata* sp. nov. **E** ms09153 **F** ms09154 **G** ms09155 *Bactrocerabryoniae* (Tryon) **H** ms01515 **I** ms01516 **J** ms07717.

### Bactrocera (Parazeugodacus) kolombangarae

Taxon classificationAnimaliaDipteraTephritidae

Leblanc & Doorenweerd sp. nov.

43C04513-B112-5FF4-9D11-8C9F0EDF596B

http://zoobank.org/DEED6917-CAC4-4647-BB4F-4454B7F7AE3C

Fig. 10A–E

#### Type material.

***Holotype*.** Solomon Islands • ♂; Kolombangara, forest; -8.0252, 157.1159; 455 m; 9–13 Apr. 2018; L. Leblanc, F. Tsatsia leg.; zingerone baited trap FFSo059. Deposited in UHIM. ***Paratypes*.** 18 males. Solomon Islands • 1 ♂; Guadalcanal forest; -9.4048, 159.8645; 144 m; 4–16 Apr. 2018; L. Leblanc, F. Tsatsia leg.; zingerone baited trap FFSo013 • 1 ♂; Kolombangara, forest; -8.0680, 157.1434; 156 m; 9–13 Apr. 2018; L. Leblanc, F. Tsatsia leg.; zingerone baited trap FFSo044 • 1 ♂; same locality and date as for preceding; -8.0563, 157.1320; 232 m; trap FFSo046 • 2 ♂; same locality and date as for preceding; -8.0512, 157.1287; 263 m; trap FFSo047; molecular vouchers UHIM.ms08663, UHIM.ms08664 • 1 ♂; same locality and date as for preceding; -8.0479, 157.1262; 267 m; trap FFSo048 • 1 ♂; same locality and date as for preceding; -8.0364, 157.1186; 331 m; trap FFSo050 • 1 ♂; same locality and date as for preceding; -8.0297, 157.1166; 403 m; trap FFSo055 • 1 ♂; same locality and date as for preceding; -8.0273, 157.1160; 433 m; trap FFSo057 • 1 ♂; same locality and date as for preceding; -8.0260, 157.1156; 446 m; trap FFSo058 • 3 ♂; same locality and date as for preceding; -8.0238, 157.1157; 464 m; trap FFSo060 • 1 ♂; same locality and date as for preceding; -8.015, 157.1143; 523 m; trap FFSo068 • 1 ♂; same locality and date as for preceding; -8.0331, 157.1081; 325 m; trap FFSo071 • 1 ♂; same locality and date as for preceding; -8.0339, 157.1129; 245 m; trap FFSo073 • 2 ♂; same locality and date as for preceding; -8.0328, 157.1164; 356 m; trap FFSo075. Nine of the paratypes are deposited at UHIM, five at WFBM, three at USNM, and one at BSI.

#### Differential diagnosis.

**Figure 10.**
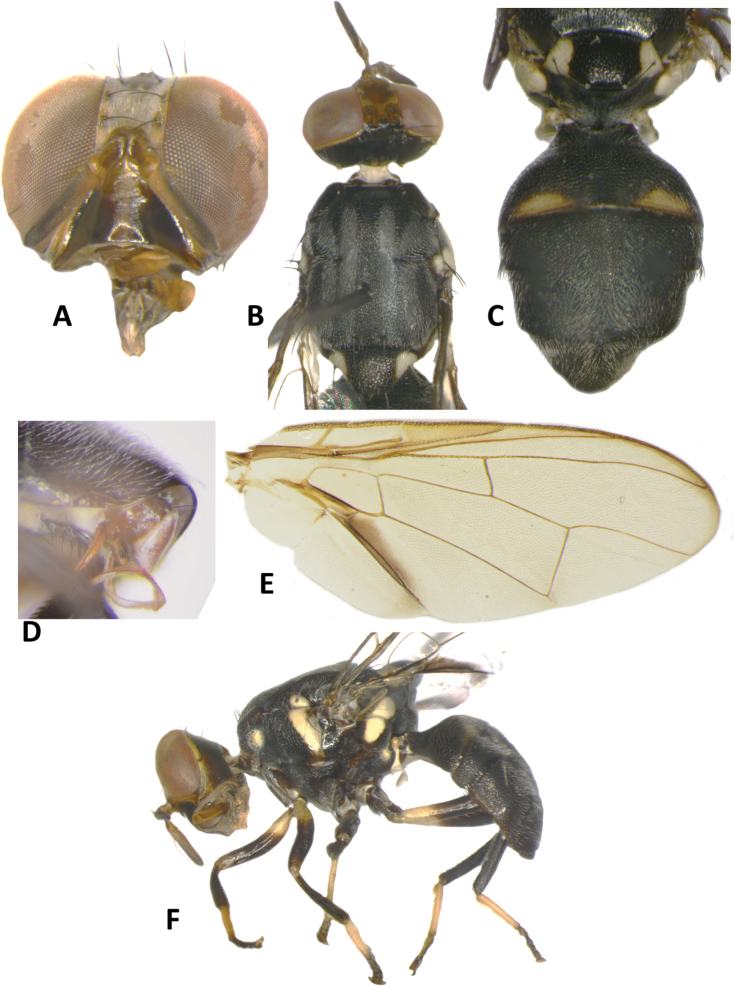
*Bactrocerakolombangarae* sp. nov. **A** head **B** head and scutum **C** abdomen **D** male genitalia **E** wing **F** lateral view.

**Figure 11.**
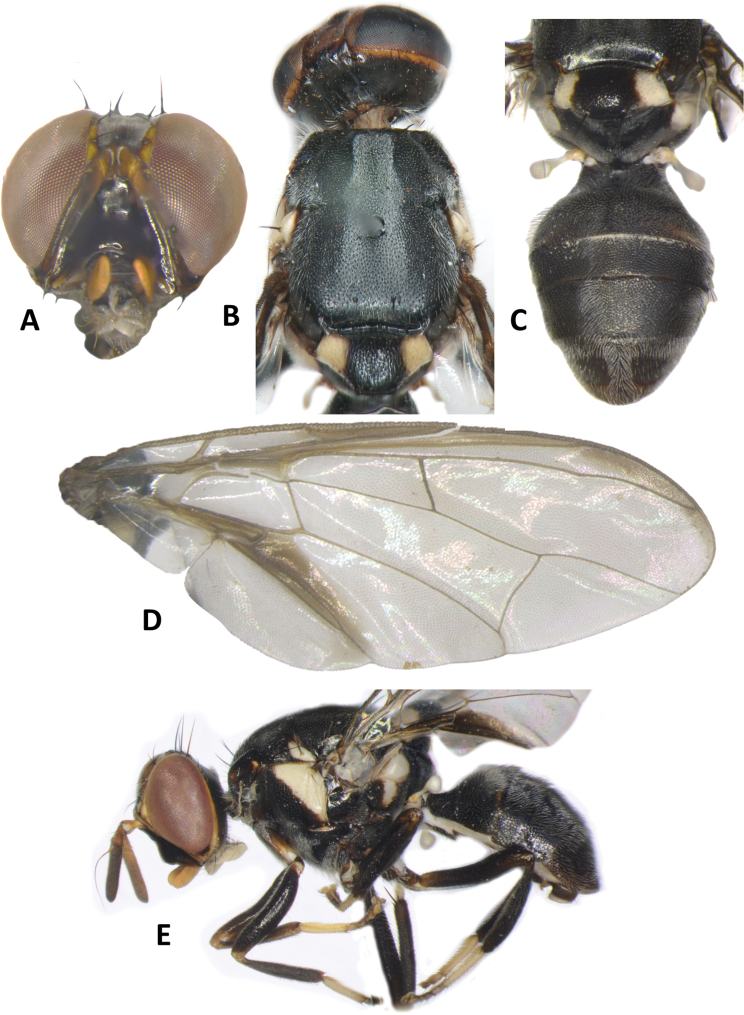
*Bactroceramorula* Drew **A** head **B** head and scutum **C** abdomen **D** wing **E** lateral view.

*Bactrocerakolombangarae* appears similar to *B.morula* (Fig. 11), but has two pairs of setae on the scutellum, a narrow anepisternal stripe, and the costal band very narrow and faint beyond the apex of R_2+3_ (Fig. 10). It is also similar to B. (Parazeugodacus) abbreviata (Hardy), a species from Southeast Asia. Unlike *B.kolombangarae*, *B.abbreviata* has yellow femora, very short lateral postsutural vitta, and orange-brown medially on abdomen tergites III–V.

#### Molecular diagnosis.

We obtained two COI sequences (UHIM.ms08663, 4) that group with other members of subgenus Parazeugodacus in the maximum likelihood tree (Suppl. material 1: Fig. S1). The sequences are closest to *Bactrocerapendleburyi* (Perkins) [N = 11] but at a distance of 3.62%, *B.abbreviata* [N = 29] and *B.morula* [N = 3] are separated with larger distances.

#### Description of adult.

**Male. *Head*** (Fig. 10A). Height 1.46 ± 0.11 (SD) (1.30–1.70) mm. Frons, of even width, 0.71 ± 0.06 (0.63–0.83) mm long and 1.57 ± 0.08 (1.46–1.77) times as long as broad; dark fulvous and frequently fuscous around orbital setae and on anteromedial hump; latter covered by short red-brown microtrichia; three pairs of black frontal setae present; lunule fulvous. Ocellar triangle black. Vertex dark fulvous with two pairs of black vertical setae. Face varying from mostly black, to lower 3⁄5 entirely black with or without traces of dark fulvous medially, and upper 2⁄5 dark fulvous, to a pair of very large spots in antennal furrows; length 0.47 ± 0.04 (0.43–0.53) mm. Gena fulvous, with fuscous subocular spot and a black seta. Occiput black and narrowly fulvous along eye margin; a row of 4–6 black postocular setae present behind eye. Antenna with scape dark fulvous, and pedicel and flagellum dark fuscous tending dark fulvous on inner surface; a strong red-brown dorsal seta on pedicel; arista fulvous basally and black apically; length of segments: 0.19 ± 0.02 (0.17–0.20) mm; 0.26 ± 0.03 (0.23–0.30) mm; 0.71 ± 0.06 (0.63–0.83) mm.

***Thorax*** (Fig. 10B). Scutum entirely black with four parallel longitudinal rows of dense silvery microtrichia along entire length and two outer rows starting before notopleural sutures. Pleural areas black. Yellow markings: notopleuron; sometimes faint marking on posterior margin of postpronotal lobe; narrow anepisternal stripe with anterior margin straight, reaching to mid distance between anterior and posterior notopleural setae dorsally; a very small spot on katepisternum below the anepisternal stripe; anterior ¼ of anatergite and anterior half of katatergite (posteriorly black). Mediotergite black. Scutellum black and narrowly yellow anterolaterally. Setae: 2 pairs scutellar; 1 pair prescutellar acrostichal; 1 pair intra-alar; 1 pair postalar; 1 pair postsutural supra-alar; 1 pair anepisternal; 2 pairs notopleural; 2 pairs scapular; all setae well developed and black.

***Legs*** (Fig. 10F). Legs black with yellow at basal 2/5 of fore and hind femora and basal 1/6 of mid femur, and yellow fore basitarsus and mid and hind tarsi. Fore femur with a row of long pale dorsal setae. Mid-tibia with an apical black spur.

***Wing*** (Fig. 10E). Length 4.9 ± 0.3 (4.5–5.6) mm; basal costal and costal cells hyaline with microtrichia in posterodistal corner of costal cell; narrow faint fuscous costal band confluent with R_2+3_, remaining narrow and ending shortly past the apex of R_2+3_; and moderately broad anal streak; remainder of wing hyaline; dense aggregation of microtrichia around A_1_ + CuA_2_; supernumerary lobe weakly developed.

***Abdomen*** (Fig. 10C, D). Oval with tergites not fused; pecten present on tergite III; posterior lobe of surstylus short; abdominal sternite V with a shallow concavity on posterior margin. Base of syntergite I+II wider than long. Tergites entirely black except for elongate creamy yellow short sublateral bands along posterior margin of tergite II. Ceromata on tergite V black. Abdominal sternites dark except for yellow sternite II.

**Female.** Unknown

#### Male attractant.

Zingerone.

#### Etymology.

This species epithet is a noun in genitive case, derived from the locality where the majority of the specimens were collected; Kolombangara Island.

#### Notes.

This species belongs to the subgenus Parazeugodacus as defined by Hancock and Drew (2015), based on morphological characters (shallow posterior concavity on male sternite V, posterior lobe of surstylus short, postpronotal seta absent, postsutural supra-alar, prescutellar acrostichal and two pairs of scutellar setae present, costal band very narrow and nearly indistinct). Its COI sequences also suggest closest affinity with other members of *Parazeugodacus* (Suppl. material 1: Fig. S1). *Bactrocerakolombangarae* was included as *B.* spnSol06 in Doorenweerd et al. (2020).

### Bactrocera (Bactrocera) quasienochra

Taxon classificationAnimaliaDipteraTephritidae

Leblanc & Doorenweerd sp. nov.

BDC868AE-C804-52B3-A8D5-69061E66B2BD

http://zoobank.org/3A13A2D0-6F79-4338-B501-887EEA24C356

Fig. 12A–E

#### Type material.

***Holotype*.** Solomon Islands • ♂; Guadalcanal, forest; -9.4064, 159.8671; 145 m; 4–16 Apr. 2018; L. Leblanc, F. Tsatsia leg.; cue-lure baited trap FFSo018; molecular voucher UHIM.ms08789. Deposited in UHIM.

#### Differential diagnosis.

**Figure 12.**
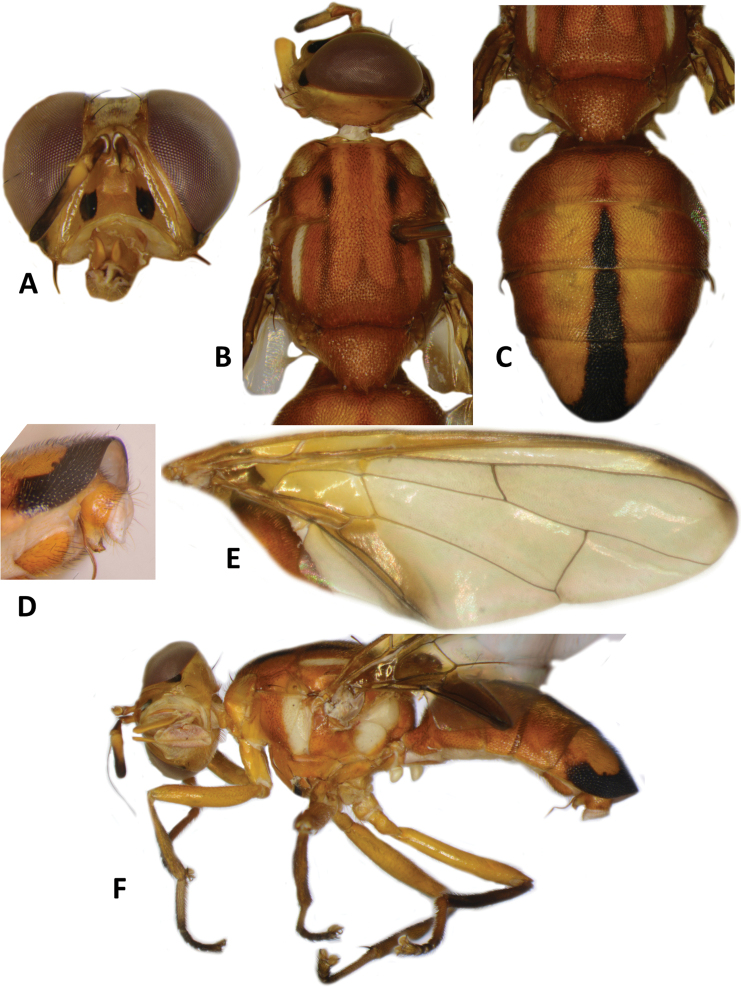
*Bactroceraquasienochra* sp. nov. **A** head **B** head and scutum **C** abdomen **D** male genitalia **E** wing **F** lateral view.

**Figure 13.**
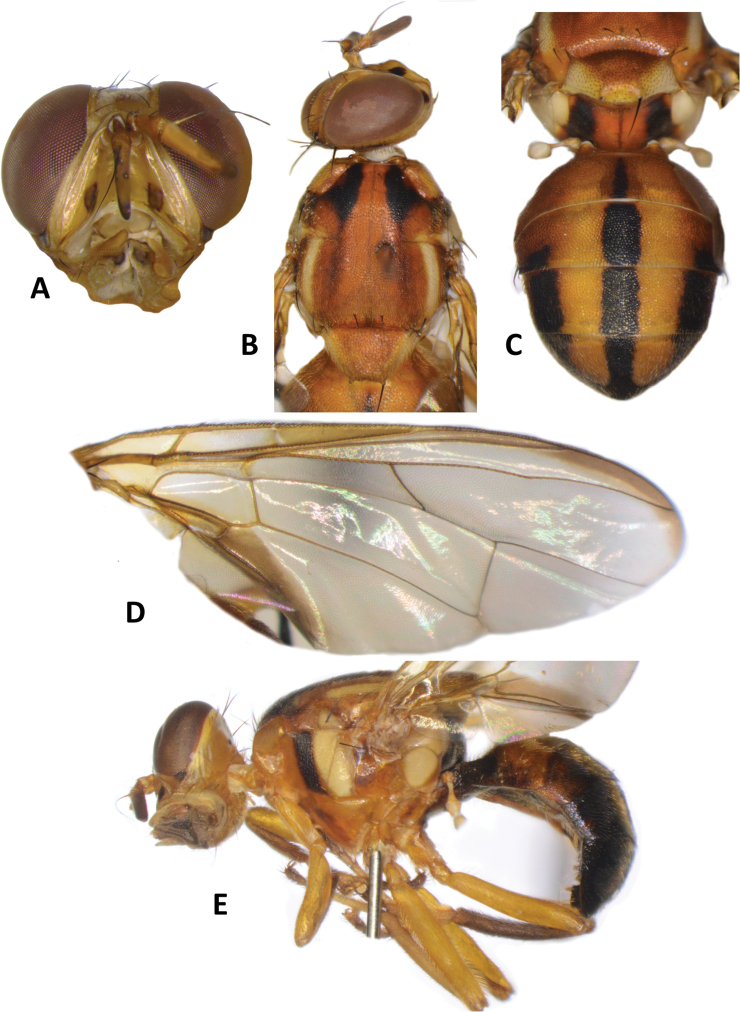
*Bactroceraenochra* (Drew) **A** head **B** head and scutum **C** abdomen **D** wing **E** lateral view.

*Bactroceraquasienochra* (Fig. 12) is similar to *B.enochra* (Drew) (Fig. 13). It differs by the absence of broad black lateral markings on abdomen tergites III–V, and the narrower lateral postsutural vitta, ending before intra-alar seta.

#### Molecular diagnosis.

We sequenced the holotype for COI, and its sequence is closest to an undescribed species from Malaysia (*B.* spMalaysia11 in Doorenweerd et al. (2020)) at 11.19% pairwise distance. The *B.quasienochra* sequence has an even greater distance to those of *B.enochra* [N = 6].

#### Description of adult.

**Male**. ***Head*** (Fig. 12A). Height 1.83 mm. Frons, of even width, 0.93 mm long and 1.56 times as long as broad; dark fulvous and narrowly fulvous anterolaterally; anteromedial hump covered by short red-brown microtrichia; three pairs of dark fuscous frontal setae present; lunule fulvous. Ocellar triangle black. Vertex fulvous with two pairs of dark fuscous vertical setae. Face fulvous with a pair of large oval black spots in antennal furrows; length 0.53 mm. Gena fulvous, with large dark fuscous subocular spot and a red-brown seta. Occiput fulvous and dark fulvous behind vertex; row of postocular setae weakly developed, with ca. four nearly indistinct setae. Antenna with scape and pedicel dark fulvous and flagellum fulvous with lateral surface and inner apical half dark fuscous; a strong fulvous dorsal seta on pedicel; arista fulvous basally and black distally; length of segments: 0.27 mm; 0.30 mm; 0.87 mm.

***Thorax*** (Fig. 12B). Scutum orange-brown with two short sublateral dark fuscous markings anterior to notopleural suture, and continued posteriorly as parallel lines formed by black microtrichia. Pleural areas orange-brown. Notopleuron light fulvous. Yellow markings: posterior half of postpronotal lobe (anteriorly orange-brown); narrow paired parallel-sided lateral postsutural vitta, slightly tapered posteriorly and ending before intra-alar seta; moderately broad anepisternal stripe with anterior margin straight, reaching to mid distance between anterior and posterior notopleural setae dorsally; anterior ⅔ anatergite and katatergite (posteriorly orange-brown). Mediotergite orange-brown. Scutellum orange-brown, and yellow on anterolateral surface and ventrally. Setae: 1 pair scutellar; prescutellar acrostichal absent; 1 pair intra-alar; 1 pair postalar; 1 pair postsutural supra-alar; 1 pair anepisternal; 2 pairs notopleural; 1 pair scapular (lateral position); all setae well developed and fuscous.

***Legs*** (Fig. 12F). Legs entirely fulvous with hind tibia tending fuscous on dorsal surface. Fore femur with a row of long fulvous dorsal setae. Mid-tibia with an apical black spur.

***Wing*** (Fig. 12E). Length 6.7 mm; basal costal and costal cells fuscous with microtrichia in posterodistal corner of costal cell; narrow fuscous costal band confluent with R_2+3_, not expanded at apex, and ending mid distance between apex of R_4+5_ and medial vein, and broad fuscous anal streak; remainder of wing hyaline; dense aggregation of microtrichia around A_1_ + CuA_2_; supernumerary lobe weakly developed.

***Abdomen*** (Fig. 12C, D). Elongate-oval with tergites not fused; pecten present on tergite III; posterior lobe of surstylus short; abdominal sternite V with a deep concavity on posterior margin. Base of syntergite I+II wider than long. All tergites orange-brown with a medial longitudinal black stripe gradually broadened from base of tergite III and extended apically along the entire lateral margins of tergite V except their bases. Ceromata on tergite V indistinct from abdomen orange-brown color. Abdominal sternites fulvous.

**Female.** Unknown

#### Male attractant.

Cue-lure.

#### Etymology.

The species name is a noun in apposition, derived from the Latin adverb *quasi* (just as if) used in conjunction with the epithet of the species it closely resembles; *B.enochra*.

#### Notes.

*Bactroceraquasienochra* was included as *B.* spnSol03 in Doorenweerd et al. (2020).

### Bactrocera (Bactrocera) tsatsiai

Taxon classificationAnimaliaDipteraTephritidae

Leblanc & Doorenweerd sp. nov.

CDC97091-1FD9-5FC0-9D6C-B3D00B1CFA84

http://zoobank.org/8B4AC740-8648-44ED-87C8-84056641FEC4

Fig. 14A–I , 15

#### Type material.

***Holotype*.** Solomon Islands • ♂; Guadalcanal, forest; -9.4053, 159.8664; 139 m; 4–16 Apr. 2018; L. Leblanc, F. Tsatsia leg.; zingerone baited trap FFSo021. Deposited in UHIM. ***Paratypes*.** 28 males. Solomon Islands • 1 ♂ Guadalcanal, forest; -9.4041, 159.8628; 153 m; 4–16 Apr. 2018; L. Leblanc, F. Tsatsia leg.; zingerone baited trap FFSo011 • 2 ♂; same locality and date as for preceding; -9.4064, 159.8644; 167 m; trap FFSo14 • 1 ♂; same locality and date as for preceding; -9.4067, 159.8647; 167 m; trap FFSo015 • 1 ♂; same locality and date as for preceding; -9.4069, 159.8664; 153 m; trap FFSo017 • 1 ♂; same locality and date as for preceding; -9.4059, 159.8672; 133 m; trap FFSo019 • 2 ♂; same locality and date as for preceding; -9.4035, 159.8681; 85 m; trap FFSo026; molecular voucher UHIM.ms08671 • 1 ♂; Kolombangara, forest; -8.0312, 157.1160; 348 m; 9–13 Apr. 2018; L. Leblanc, F. Tsatsia leg.; zingerone baited trap FFSo053 • 3 ♂; same locality and date as for preceding; -8.0297, 157.1166; 403 m; trap FFSo055 • 1 ♂; same locality and date as for preceding; -8.0283, 157.1159; 426 m; trap FFSo056 • 3 ♂; same locality and date as for preceding; -8.0218, 157.1150; 491 m; trap FFSo062 • 2 ♂; same locality and date as for preceding; -8.0200, 157.1143; 508 m; trap FFSo063 • 2 ♂; same locality and date as for preceding; -8.0190, 157.1133; 520 m; trap FFSo064 • 1 ♂; same locality and date as for preceding; -8.0181, 157.1129; 518 m; trap FFSo065 • 1 ♂; same locality and date as for preceding; -8.0181, 157.1134; 526 m; trap FFSo066 • 1 ♂; same locality and date as for preceding; -8.0157, 157.1118; 506 m; trap FFSo067 • 1 ♂; same locality and date as for preceding; -8.0150, 157.1143; 523 m; trap FFSo068 • 1 ♂; same locality and date as for preceding; -8.0327, 157.1159; 333 m; trap FFSo070 • 2 ♂; same locality and date as for preceding; -8.0356, 157.1193; 352 m; trap FFSo077 • 1 ♂; same locality and date as for preceding; -8.0357, 157.1200; 352 m; trap FFSo078. Fifteen of the paratypes are deposited at UHIM, seven at WFBM, four at USNM, and two at BSI.

#### Differential diagnosis.

The broad orange-brown medial marking on the scutum uniquely defines *Bactroceratsatsiai* within the genus, where all other species have either a yellow mark or no mark.

#### Molecular diagnosis.

We obtained two COI sequences that are most similar to *Bactrocerahantanae* Tsuruta & White but at 10.79% pairwise distance.

#### Description of adult.

**Figure 14.**
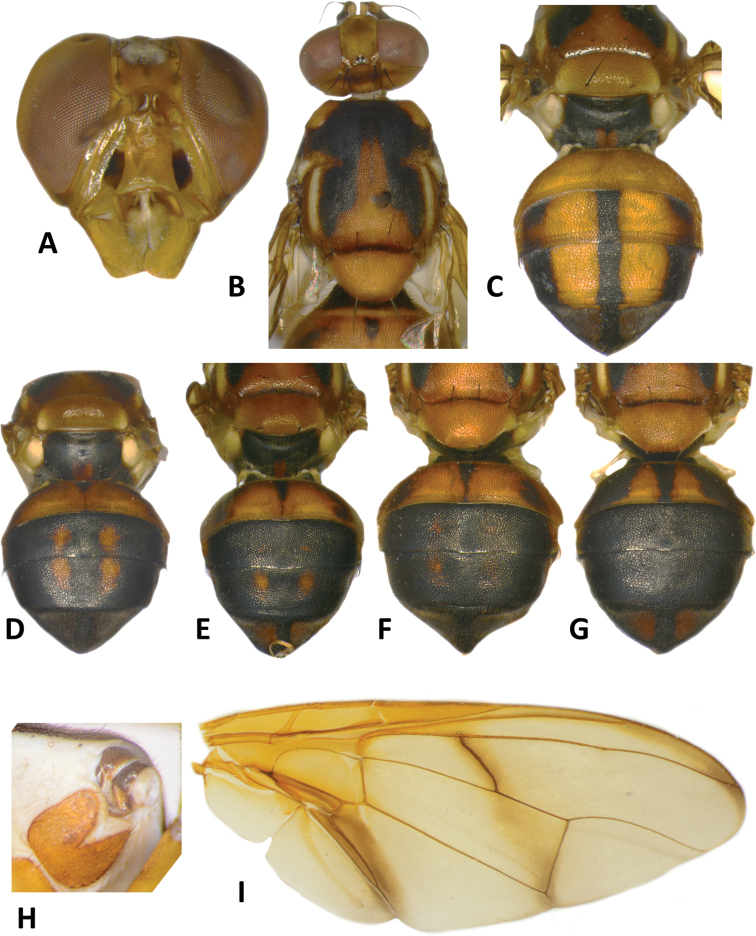
*Bactroceratsatsiai* sp. nov. **A** head **B** head and scutum **C–G** abdomen variants **H** male genitalia **I** wing.

**Male. *Head*** (Fig. 14A). Height 2.00 ± 0.09 (SD) (1.87–2.13) mm. Frons, of even width, 0.99 ± 0.04 (0.93–1.07) mm long and 1.56 ± 0.06 (1.47–1.63) times as long as broad; fulvous with red-brown microtrichia on anteromedial hump; three pairs of black frontal setae present; lunule yellow. Ocellar triangle black. Vertex fuscous with two pairs of black vertical setae. Face fulvous with a pair of large oval black spots in antennal furrows; length 0.60 ± 0.04 (0.53–0.67) mm. Gena fulvous, with small dark fuscous subocular spot and a black seta. Occiput fulvous; a row of 6–9 black postocular setae present behind eye. Antenna with scape and pedicel fulvous and flagellum fuscous with fulvous on inner surface; a strong black dorsal seta on pedicel; arista fulvous basally and black distally; length of segments: 0.25 ± 0.03 (0.20–0.30) mm; 0.32 ± 0.03 (0.27–0.37) mm; 0.87 ± 0.05 (0.80–0.93) mm.

***Thorax*** (Fig. 14B). Scutum dark fuscous with orange-brown ventral to and narrowly anterior to lateral postsutural vitta, narrowly englobing notopleural suture, between postpronotal lobe and notopleuron, and as a medial band starting before notopleural suture and enlarged posteriorly to cover entire posterior margin region of scutum. Pleural areas black except orange-brown anepisternum and proepisternum. Yellow markings: postpronotal lobe (or may be anteriorly to entirely orange-brown), notopleuron; moderately broad paired parallel-sided lateral postsutural vitta ending at intra-alar seta posteriorly; moderately broad anepisternal stripe with anterior margin slightly convex, reaching to mid distance between anterior and posterior notopleural setae dorsally; a small transverse spot on katepisternum below the anepisternal stripe; anterior ¾ of anatergite and katatergite (posteriorly black). Mediotergite black. Scutellum orange-brown, and yellow ventrally and narrowly on dorsolateral surface. Setae: 1 pair scutellar; 1 pair prescutellar acrostichal; 1 pair intra-alar; 1 pair postalar; 1 pair postsutural supra-alar; 1 pair anepisternal; 2 pairs notopleural; 2 pairs scapular; all setae well developed and black.

**Figure 15.**
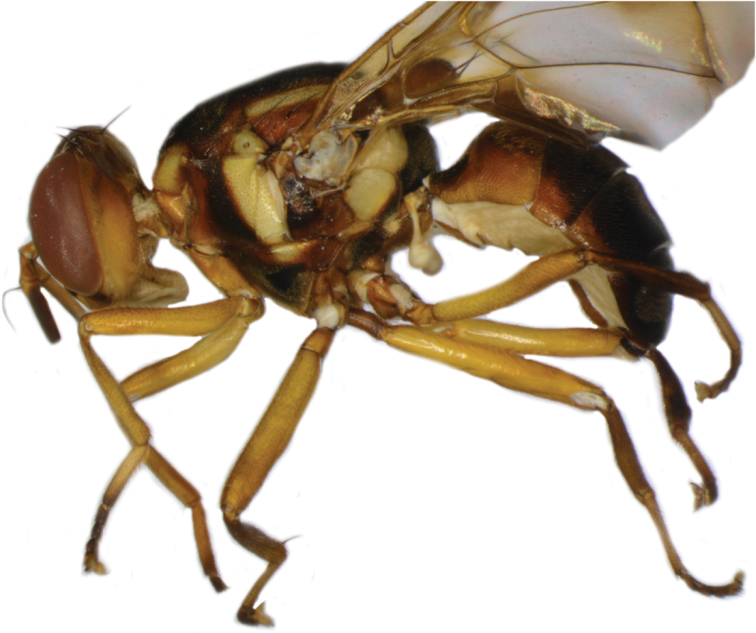
*Bactroceratsatsiai* sp. nov. lateral view.

***Legs*** (Fig. 15). All legs entirely fulvous with hind femur and fore tarsomeres II–IV fuscous. Fore femur with a row of long pale dorsal setae. Mid-tibia with an apical black spur.

***Wing*** (Fig. 14I). Length 7.1 ± 0.3 (6.6–7.5) mm; basal costal and costal cells fuscous with microtrichia in posterodistal corner of costal cell; light fuscous costal band confluent with R_2+3_, not expanded at apex and ending mid distance between apex of R_4+5_ and medial vein, a diffuse broad fuscous cross band along r-m crossvein, continuing in straight line through discal medial (dm) cell and reaching wing margin at level of CuA_1_, and a broad fuscous anal streak; remainder of wing hyaline; dense aggregation of microtrichia around A_1_ + CuA_2_; supernumerary lobe moderately developed.

***Abdomen*** (Fig. 14C–H). Oval with tergites not fused; pecten present on tergite III; posterior lobe of surstylus short; abdominal sternite V with a deep concavity on posterior margin. Base of syntergite I+II wider than long. Syntergite I+II with tergite I black and tergite II orange-brown with or without a small basal black triangular and two small sublateral black markings. Tergites III–V orange-brown with broad medial longitudinal black stripe reaching apex of tergite V and extended apically along entire lateral margins of tergite V, and two broad sublateral stripes covering tergite III (may be interrupted on that tergite) and continuing on tergite IV and along lateral margins on tergite V. Dark marking variable and may cover almost all of tergites III–V (Fig. 14C–G). Ceromata on tergite V dark fuscous. Abdominal sternites fulvous.

**Female.** Unknown

#### Male attractant.

Zingerone.

#### Etymology.

The epithet *tsatsiai* is a noun in genitive case, referring to the personal name Francis Tsatsia, a long-time colleague, friend, co-author of the present publication, and currently the director of Biosecurity Solomon Islands.

#### Notes.

*Bactroceratsatsiai* was included as *B.* spnSol05 in Doorenweerd et al. (2020).

### Bactrocera (Bactrocera) vargasi

Taxon classificationAnimaliaDipteraTephritidae

Leblanc & Doorenweerd sp. nov.

D016B3BF-ABC7-5133-A17E-DC86D5661ABD

http://zoobank.org/BC8E46E7-1917-412C-AF67-7A487BDEFAFE

Fig. 16A–F

#### Type material.

***Holotype*.** Solomon Islands • ♂; Kolombangara, forest; -8.0563, 157.1320; 232 m; 9–13 Apr. 2018; L. Leblanc, F. Tsatsia leg.; zingerone baited trap FFSo046. Deposited in UHIM. ***Paratypes*.** 42 males. Solomon Islands • 2 ♂; Guadalcanal, forest; 4–16-iv-2018; L. Leblanc, F. Tsatsia leg.; -9.4041, 159.8628; 153 m; zingerone trap FFSo011 • 2 ♂; same locality and date as for preceding; -9.4045, 159.8644; 142 m; trap FFSo012 • 2 ♂; same locality and date as for preceding; -9.4048, 159.8645; 144 m; trap FFSo013 • 2 ♂; same locality and date as for preceding; -9.4064, 159.8644; 167 m; trap FFSo14 • 3 ♂; same locality and date as for preceding; -9.4067, 159.8647; 167 m; trap FFSo015; molecular vouchers UHIM.ms08665, UHIM.ms08666, UHIM.ms08667 • 2 ♂; same locality and date as for preceding; -9.4069, 159.8664; 153 m; trap FFSo017 • 4 ♂; same locality and date as for preceding; -9.4064, 159.8671; 145 m; trap FFSo018 • 2 ♂; same locality and date as for preceding; -9.4059, 159.8672; 133 m; trap FFSo019 • 2 ♂; same locality and date as for preceding; -9.4055, 159.8665; 145 m; trap FFSo020 • 1 ♂; same locality and date as for preceding; -9.4053, 159.8664; 139 m; trap FFSo021 • 3 ♂; same locality and date as for preceding; -9.4040, 159.8652; 125 m; trap FFSo023 • 3 ♂; same locality and date as for preceding; -9.4038, 159.8646; 103 m; trap FFSo024 • 1 ♂; same locality and date as for preceding; -9.4039, 159.8673; 103 m; trap FFSo025 • 2 ♂; same locality and date as for preceding; -9.4035, 159.8681; 85 m; trap FFSo026 • 2 ♂; same locality and date as for preceding; -9.4026, 159.8695; 57 m; trap FFSo027 • 1 ♂; same locality and date as for preceding; -9.400, 159.8700; 50 m; trap FFSo029 • 2 ♂; Kolombangara, forest; -8.0563, 157.1320; 232 m; 9–13 Apr. 2018; L. Leblanc, F. Tsatsia leg.; zingerone baited trap FFSo046 • 2 ♂; same locality and date as for preceding; -8.0479, 157.1262; 267 m; trap FFSo048 • 1 ♂; same locality and date as for preceding; -8.0306, 157.1168; 389 m; trap FFSo054 • 1 ♂; same locality and date as for preceding; -8.0252, 157.1159; 455 m; trap FFSo059 • 1 ♂; same locality and date as for preceding; -8.0328, 157.1164; 356 m; trap FFSo075 • 1 ♂; same locality and date as for preceding; -8.0395, 157.1237; 308 m; trap FFSo079. 29 of the paratypes are deposited at UHIM, seven at WFBM, four at USNM, and two at BSI.

#### Differential diagnosis.

**Figure 16.**
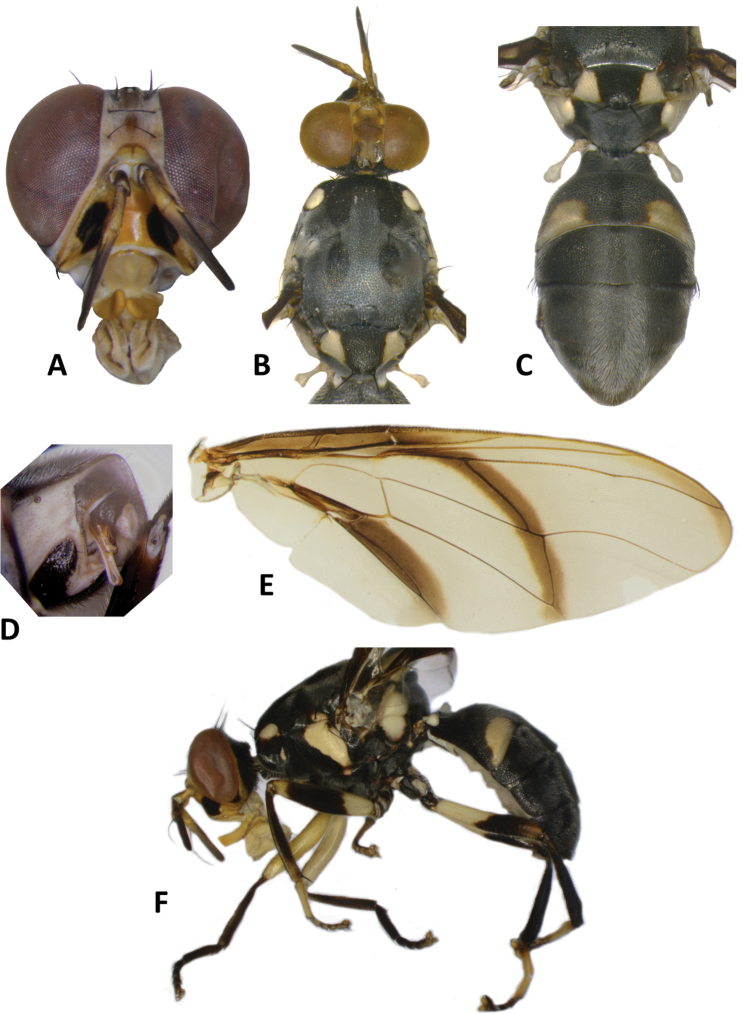
*Bactroceravargasi* sp. nov. **A** head **B** head and scutum **C** abdomen **D** male genitalia **E** wing **F** lateral view.

**Figure 17.**
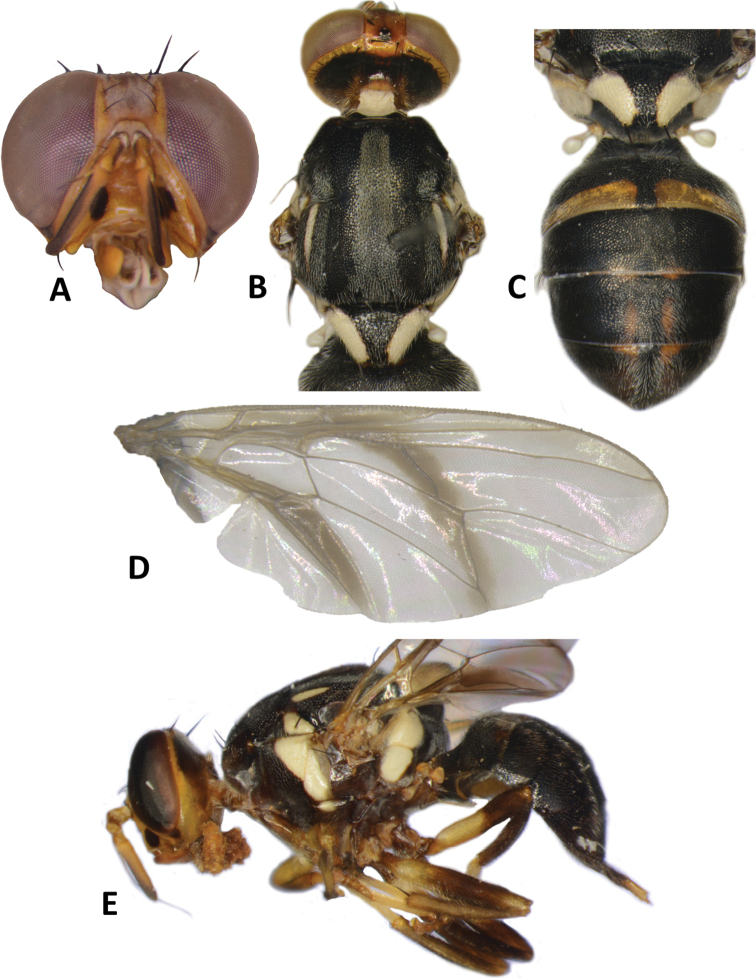
*Bactrocerafrauenfeldi* (Schiner) **A** head **B** head and scutum **C** abdomen, male **D** wing **E** lateral view, female.

The overall appearance and specifically the wing of *B.vargasi* (Fig. 16E) is very similar to that of *B.frauenfeldi* (Schiner) (Fig. 17) [Solomon Island populations], *B.trilineola* Drew and *B.parafrauenfeldi* Drew [all three are members of the morphological *B.frauenfeldi* complex], but *B.vargasi* differs from *B.trilineola* and *B.parafrauenfeldi* in having a nearly entirely black abdomen (Fig. 16), and can be separated from *B.frauenfeldi* in lacking lateral postsutural yellow vitta.

#### Molecular diagnosis.

We sequenced three specimens which have COI sequences closest to *B.quasiinfulata* Drew & Romig at 7.24% minimum pairwise distance. The maximum intraspecific distance is 1.2%. Sequences of the morphologically similar *B.frauenfeldi* and *B.trilineola* were also included in the reference dataset but are highly dissimilar to *B.vargasi* with >8% pairwise distance. *Bactroceraparafrauenfeldi* was not included in the reference set but is presumed to be closely related to *B.trilineola* (Drew 1989).

#### Description of adult.

**Male. *Head*** (Fig. 16A). Height 1.61 ± 0.22 (SD) (1.33–1.93) mm. Frons, of even width, 0.83 ± 0.08 (0.67–0.93) mm long and 1.63 ± 0.11 (1.43–1.85) times as long as broad; fulvous and narrowly yellow along eye margin; anteromedial hump covered by short red-brown microtrichia; three pairs of black frontal setae present; lunule yellow. Ocellar triangle black. Vertex black with yellow spot behind ocellar triangle and two pairs of black vertical setae. Face fulvous with a pair of very large circular black spots in antennal furrows; length 0.51 ± 0.06 (0.43–0.63) mm. Gena fulvous, with small dark fuscous subocular spot and a red-brown seta. Occiput black and narrowly fulvous along eye margin; a row of 6–9 black postocular setae present behind eye. Antenna with scape and pedicel fulvous and flagellum dark fuscous tending dark fulvous on inner surface; a strong red-brown dorsal seta on pedicel; arista fulvous basally and black distally; length of segments: 0.31 ± 0.03 (0.27–0.33) mm; 0.36 ± 0.03 (0.33–0.40) mm; 0.88 ± 0.09 (0.73–1.00) mm.

***Thorax*** (Fig. 16B). Scutum entirely black with dense silvery microtrichia on all scutum except two broad parallel longitudinal shining black areas interrupted at level of notopleural suture. Pleural areas black. Yellow markings: posterior half of postpronotal lobe (anteriorly fuscous); notopleuron; moderately broad anepisternal stripe with anterior margin convex, reaching to mid distance between anterior and posterior notopleural setae dorsally; a very small transverse spot on katepisternum below the anepisternal stripe; anterior 3⁄5 of anatergite and katatergite (posteriorly black). Mediotergite black. Scutellum broadly black medially and yellow laterally. Setae: 1 pair scutellar; 1 pair prescutellar acrostichal; 1 pair intra-alar; 1 pair postalar; 1 pair postsutural supra-alar; 1 pair anepisternal; 2 pairs notopleural; 2 pairs scapular; all setae well developed and black.

***Legs*** (Fig. 16F). Legs black with yellow fore femur, basal 2⁄5 of mid and hind femur, and mid and hind tarsi. Fore femur with a row of long pale dorsal setae. Mid-tibia with an apical black spur.

***Wing*** (Fig. 16E). Length 6.2 ± 0.6 (5.3–6.9) mm; basal costal and costal cells dark fuscous with microtrichia covering both cells; faint narrow fuscous costal band confluent with R_2+3_, remaining narrow to end shortly past the apex of R_2+3_; dark fuscous straight band across r-m and dm-cu veins and reaching wing margin; broad dark fuscous anal streak; remainder of wing hyaline; dense aggregation of microtrichia around A_1_ + CuA_2_; supernumerary lobe weakly developed.

***Abdomen*** (Fig. 16C). Oval with tergites not fused; pecten present on tergite III; posterior lobe of surstylus short; abdominal sternum V with a deep concavity on posterior margin. Base of syntergite I+II wider than long. Tergites entirely black with yellow lateral bands along posterior margin of tergite II. Ceromata on tergite V black. Abdominal sternites black.

**Female.** Unknown.

#### Etymology.

We proudly name this species to honor the famous fruit fly ecologist Roger I. Vargas (1947–2018) (Stark et al. 2018). The species name *vargasi* is a noun in genitive case. Roger and LL collaborated extensively on projects during years spent in the South Pacific Islands. Roger brought LL to Hawaii in 2003 to continue working on fruit flies, and he secured funding and provided guidance that allowed LL to obtain a PhD title in 2010.

#### Male attractant.

Zingerone.

#### Notes.

*Bactroceravargasi* was included as *B.* spnSol07 in Doorenweerd et al. (2020).

### Key to Dacine fruit fly species of Solomon Islands

This is a modified version of the key published by Drew and Romig (2001). We include for each species subgenus assignment and information on male lure attraction and host fruit (after Leblanc et al. 2012), whenever known.

**Table:** 

1	Elongate large wasp-like fly; antenna longer than height of face; abdomen elongate and petiolate (base of syntergite I+II longer than wide), with a pronounced hump on tergite V in lateral view (unique to that species) (Fig. 18A–D) (cue-lure) (pest of cucurbit fruits)	**Dacus (Mellesis) solomonensis (Malloch)**
–	More compact typical fly; antenna shorter than height of face; abdomen oval and not petiolate (base of syntergite I+II wider than long), and never with a hump on tergite V	**2**
2	Wing without complete costal band, with large faint light fuscous spot covering apex, and a swelling (bulla) in CuA_2_ cell; medial postsutural vitta large and triangular (Fig. 18 E–H) (bred from *Terminalia catappa* and *Gnetumgnemon*)	**males of B. (Bulladacus) penefurva Drew**
–	Wing with complete costal band, although sometimes noticeably paler beyond apex of R_1_, with marking (when present) not as large spot at apex, and bulla absent (except in males of *B.pacificae*); medial postsutural vitta present or absent	**3**
3	Wing membrane with infuscation in addition to costal band and anal streak (this may be narrow infuscation on one or both crossveins) (Figs 18K–24F)	**4**
–	Wing membrane colorless or lightly infuscated, except for costal band and anal streak (Figs 24I–28L)	**31**
4	Scutum with medial postsutural vitta (Figs 18I–20A)	**5**
–	Scutum without medial postsutural vitta (Figs 20D–24D)	**11**
5	Prescutellar acrostichal seta absent; postsutural supra-alar seta present or absent	**6**
–	Prescutellar acrostichal and postsutural supra-alar seta present	**8**
6	Postsutural supra-alar seta absent; abdomen fulvous with broad dark fuscous lateral stripes on tergites III–V (Fig. 18I–K) (zingerone)	**B. (Tetradacus) pagdeni (Malloch)**
–	Postsutural supra-alar seta present; abdomen with black spot on tergite V (Fig. 18M) or with narrow medial and lateral stripes (Fig. 19B)	**7**
7	Postpronotal lobe and notopleuron joined by lateral yellow band; wing with narrow pale infuscation along crossveins r-m and dm-cu; abdominal tergites entirely red-brown except for black spot in center of tergite V (Fig. 18L–N) (cue-lure)	**Z. (Zeugodacus) univittatus (Drew)**
–	Postpronotal lobe and notopleuron not joined by yellow band; wing with fuscous tint throughout, broad fuscous costal band to R_4+5_, narrow infuscation along r-m crossvein and broad infuscation along dm-cu crossvein; abdominal tergites III–V with narrow medial and lateral longitudinal black stripes (Fig. 19A–C) (cue-lure)	**Z. (Javadacus) hamaceki Drew & Romig**
8	Scutum glossy black; Z-shaped fuscous pattern across wing (Fig. 19D–F) (cue-lure)	**Z. (Zeugodacus) amoenus (Drew)**
–	Scutum basically red-brown; wing with infuscation on one or both crossveins, but not a Z-shaped pattern	**9**
9	Scutum red-brown with small dark markings and a very narrow medial postsutural vitta; scutellum with one or two pairs of setae (Fig. 19G–I) (cue-lure, zingerone) (pest of cucurbit flowers and fruits)	**Z. (Javadacus) cucurbitae (Coquillett)**
–	Scutum entirely red-brown or red-brown with large dark markings and a broader medial postsutural vitta (Figs 19J, 20A); scutellum with two pairs of setae	**10**
10	Wing with infuscation on dm-cu crossvein only; scutum entirely red-brown; abdominal tergites III–V red-brown without a distinct dark ‘T’-shaped pattern (Fig. 19J–L)	**Z. (Javadacus) fuscipennulus (Drew & Romig)**
–	Wing with infuscation on both crossveins; scutum red-brown with large black markings; abdominal tergites III–V red-brown with a black ‘T’-shaped pattern (Fig. 20A–C) (cue-lure)	**Z. (Javadacus) abdoangustus (Drew)**
11	Infuscation on wing on one crossvein only (Fig. 20F, J, N)	**12**
–	Infuscation on wing more extensive, as a very broad pattern across most of membrane, a recurved band, or one or more transverse bands	**14**
12	Lateral postsutural vitta very short and narrowing posteriorly to end well before intra-alar seta (Fig. 20D–F) (methyl eugenol)	**B. (Bactrocera) melanogaster (Drew)**
–	Lateral postsutural vitta broad, parallel sided (or with only a slight narrowing posteriorly) and ending at intra-alar seta	**13**
13	Anepisternal stripe reaching to postpronotal lobe dorsally; abdominal tergites III–V red-brown with a black ‘T’-shaped and broad lateral black margins (Fig. 20G–I) (cue-lure) (bred from *Alpiniapurpurata*)	**B. (Bactrocera) phaea (Drew)**
–	Anepisternal stripe reaching to anterior notopleural seta dorsally; abdominal tergites mostly black (Fig. 24K–N) (methyl eugenol)	**B. (Bactrocera) neonigrita Drew**
14	Scutellum with a black triangular dorsal marking or with an apical dark spot	**15**
–	Scutellum yellow or orange-brown, except for a narrow dark basal band	**20**
15	Costal band pale and indistinct beyond apex of R_1_; a narrow transverse fuscous band across wing	**16**
–	Costal band distinct for entire length; infuscation across wing as a single band or a recurved band	**17**
16	Lateral postsutural vitta present; abdomen orange-brown with a broad medial and two broad lateral black bands along tergites III–V (Fig. 17A–E) (cue-lure, zingerone) (polyphagous fruit pest bred from 28 host species in Solomon Islands)	**B. (Bactrocera) frauenfeldi (Schiner)**
–	Lateral postsutural vitta absent; abdomen entirely black except yellow lateral bands along posterior margin of tergite II (Fig. 16A–F) (zingerone)	**B. (Bactrocera) vargasi sp. nov.**
17	Infuscation across wing as a recurved band (Fig. 21C, F)	**18**
–	Infuscation across wing as a single band (Figs 21I, 22C)	**19**
18	Single U-shaped band across wing (Fig. 21A–C) (methyl eugenol)	**B. (Bactrocera) reclinata Drew**
–	Broad recurved band across center of wing and a narrow transverse band across apex (Fig. 21D–F) (cue-lure)	**B. (Bactrocera) longicornis Macquart**
19	Scutellum with a broad medial longitudinal black stripe; postpronotal lobe yellow except for anterior third dark fuscous to black; postpronotal lobe and notopleuron not joined by a yellow band; lateral postsutural vitta short and narrow; abdomen tergites III–V orange-brown with small irregularly shaped sublateral markings and a narrow medial longitudinal stripe (Fig. 21G–I) (cue-lure)	**B. (Bactrocera) hollingsworthi Drew & Romig**
–	Scutellum yellow with at most an apical dark spot; postpronotal lobe yellow; postpronotal lobe and notopleuron joined by a narrow yellow band; lateral postsutural vitta well developed; abdomen tergites III–V orange-brown with a black medial longitudinal stripe and well defined black lateral markings (Fig. 22A–C) (cue-lure)	**B. (Bactrocera) unitaeniola Drew & Romig**
20	Wing with three broad transverse fuscous bands (Fig. 22D–F) (methyl eugenol) (pest of breadfruit and jackfruit)	**B. (Bactrocera) umbrosa (Fabricius)**
–	Wing not so marked	**21**
21	Postpronotal lobe and notopleuron joined by a broad yellow band (Fig. 22G–I) (cue-lure)	**B. (Bactrocera) unifasciata (Malloch)**
–	Postpronotal lobe and notopleuron not joined by a yellow band	**22**
22	Abdomen tergites III–V entirely black	**23**
–	Abdomen tergites III–V orange-brown with dark markings	**25**
23	Wing membrane almost entirely fuscous; abdomen tergite II largely fulvous, contrasting with the black tergites III–V (Fig. 22J–L) (methyl eugenol)	**B. (Bactrocera) pepisalae (Froggatt)**
–	Wing membrane colorless with distinct fuscous markings; abdomen entirely black, or with at most an orange-brown band along posterior margin of tergite II	**24**
24	r-m crossvein strongly oblique; broad dark fuscous band across wing from costal band to hind margin, enclosing both crossveins; legs entirely fulvous (Fig. 23A–C) (methyl eugenol)	**B. (Bactrocera) obliquivenosa Drew & Romig**
–	r-m crossvein not oblique; transverse fuscous band across wing broad and covering more than outer half of discal medial cell; legs fulvous with apical half of mid and hind femur black (Fig. 23D–F) (methyl eugenol)	**B. (Bactrocera) biarcuata (Walker)**
25	Z-shaped fuscous pattern across wing	**26**
–	Single fuscous band of variable shape across wing	**27**
26	Lateral postsutural vitta short and tapering posteriorly; wing markings dark fuscous; lateral and medial longitudinal black stripes on abdominal tergites III–V sometimes joined across base of tergite III (Fig. 23G–I) (cue-lure)	**B. (Bactrocera) nigrescentis (Drew)**
–	Lateral postsutural vitta broad, parallel sided and reaching to intra-alar seta; wing markings pale fuscous; lateral and medial longitudinal black stripes on abdominal tergites III–V not joined (Fig. 23J–L) (cue-lure) (bred from *Pycnarrhenaozantha* in Vanuatu)	**B. (Bactrocera) redunca (Drew)**
27	Large species (body length 11 mm or more); transverse fuscous band across wing broad and covering more than outer half of discal medial cell (Fig. 24A–C) (methyl eugenol)	**B. (Bactrocera) confluens (Drew)**
–	Moderately sized species (body length 9 mm or less); transverse fuscous band across wing of medium width, covering outer third of discal medial cell	**28**
28	Wing crossband dark fuscous and broad; costal band confluent with R_4+5_ and greatly expanded at apex of wing (Fig. 24D–F) (cue-lure) (bred from *Burckela* sp.)	**B. (Bactrocera) decumana (Drew)**
–	Wing crossband light fuscous; costal band not greatly expanded at apex of wing	**29**
29	Scutum dark fuscous to black with a broad orange-brown medial stripe, starting before notopleural suture and enlarged posteriorly to cover entire posterior margin region of scutum; scutellum largely orange-brown, and yellow ventrally and narrowly on dorsolateral surface; anepisternal stripe moderately broad, reaching to mid distance between anterior and posterior notopleural setae dorsally (Figs 14A–I, 15) (zingerone)	**B. (Bactrocera) tsatsiai sp. nov.**
–	Scutum predominantly to entirely black, at most narrowly orange-brown laterally and posteriorly; scutellum yellow; anepisternal stripe broad, almost reaching to anterior notopleural seta dorsally	**30**
30	Scutum entirely black, except for yellow postpronotal lobe, notopleuron and lateral postsutural vitta; abdominal tergites III–V with moderately broad medial longitudinal black stripe and lateral black markings on tergite IV not narrowed posteriorly; fuscous crossband on wing clearly defined (Fig. 4A–E) (cue-lure)	**B. (Bactrocera) pseudodistincta (Drew)**
–	Scutum predominantly black with orange-brown laterally and posteriorly; abdominal tergites III–V with narrower medial longitudinal black stripe and lateral black markings on tergite IV narrowed posteriorly; fuscous crossband on wing diffuse (Fig. 3A–F) (cue-lure)	**B. (Bactrocera) allodistincta sp. nov.**
31	Lateral postsutural vitta absent	**32**
–	Lateral postsutural vitta present	**34**
32	Scutellum entirely yellow; abdominal tergites orange-brown with a narrow median longitudinal stripe on tergites III–V (Fig. 24G–I) (cue-lure) (bred from *Cerbera* spp and *Antiaristoxicaria* in Vanuatu)	**B. (Bactrocera) minuta (Drew)**
–	Scutellum yellow and broadly black medially; abdominal tergites black	**33**
33	Scutellum with two pairs of setae; anepisternal stripe narrow and reaching to mid distance between anterior and posterior notopleural setae dorsally (Fig. 10A–F) (zingerone)	**B. (Parazeugodacus) kolombangarae sp. nov.**
–	Scutellum with one pair of setae; anepisternal stripe broader and reaching posterior notopleural seta dorsally (Fig. 11A–E) (cue-lure)	**B. (Bactrocera) morula Drew**
34	Costal band confluent with or overlapping R_4+5_	**35**
–	Costal band not reaching to R_4+5_	**43**
35	Scutum and abdominal tergites mostly red-brown	**36**
–	Scutum black or dark fuscous with a pair of longitudinal black bands; abdominal tergites mostly black or orange-brown or red-brown with dark markings	**37**
36	Anepisternal stripe reaching to anterior notopleural seta; pecten present on male abdominal tergite III (Fig. 24J–L) (methyl eugenol) (bred from *Nauclea* sp.)	**B. (Bactrocera) naucleae Drew & Romig**
–	Anepisternal stripe ending midway between anterior margin of notopleuron and anterior notopleural seta; pecten absent from male abdominal tergite III (Fig. 25A–C) (bred from *Spondiasdulcis*)	**B. (Calodacus) hastigerina (Hardy)**
37	Abdominal tergites III–V red-brown with a black ‘T’-shaped pattern and narrow lateral dark margins; pecten absent from male abdominal tergite III (Fig. 25D–F) (bred from *Calophyllum* spp)	**B. (Calodacus) calophylli (Perkins & May)**
–	Abdominal tergites III–V mostly black or orange-brown with broad medial and longitudinal black stripes; pecten present on male abdominal tergite III	**38**
38	Abdominal tergites III–V orange-brown with broad medial and lateral longitudinal black stripes that are not joined (Fig. 25G–I) (methyl eugenol)	**B. (Bactrocera) froggatti (Bezzi)**
–	Abdominal tergites mostly black	**39**
39	Costal band overlapping R_4+5_ for entire length; abdominal tergites mostly black, with some orange-brown centrally on tergites IV and V	**40**
–	Costal band confluent with R_4+5_; abdominal tergites entirely black	**41**
40	Wing (Fig. 9E–G) with a light fuscous tinge as a broad, somewhat triangular area covering much of the middle of the wing, including the areas bordering r-m and dm-cu (Figs 6A–E, 9E–G) (cue-lure)	**B. (Bactrocera) geminosimulata sp. nov.**
–	Wing (Fig. 9A–D) without a light fuscous tinge in the area described above (Figs 7A–D, 9A–D) (cue-lure) (bred from *Cocciniagrandis*)	**B. (Bactrocera) simulata (Malloch)**
41	Microtrichia covering all of basal costal and costal cells in wing (Fig. 25J–L) (dihydroeugenol, isoeugenol) (bred from *Allophyluscobbe* (formerly *Pometiapinnata*))	**B. (Bactrocera) quadrisetosa (Bezzi)**
–	Microtrichia restricted to posterodistal corner of costal cell in wing	**42**
42	Legs mostly black; scutellum with a broad black basal band; anepisternal stripe narrow, just wider than notopleuron (Fig. 26A–C) (cue-lure)	**B. (Bactrocera) epicharis (Hardy)**
–	Legs mostly fulvous; scutellum with a narrow black basal band; anepisternal stripe reaching to anterior notopleural seta (Fig. 26D–F) (cue-lure)	**B. (Bactrocera) atrabifasciata Drew & Romig**
43	Scutellum yellow with dark markings or orange-brown and narrowly yellow laterally	**44**
–	Scutellum entirely yellow or entirely orange-brown	**49**
44	Scutellum with a dark apical spot (Fig. 26G–I) (methyl eugenol) (bred from Moraceae in Australia and *Pimelodendronamboicinum* in Papua New Guinea)	**some specimens of B. (Bactrocera) bancroftii (Tryon)**
–	Scutellum with a black or brown longitudinal marking over dorsal surface	**45**
45	Scutum and abdomen predominantly black (Fig. 26J–L) (methyl eugenol)	**B. (Bactrocera) picea (Drew)**
–	Scutum and abdomen predominantly red-brown	**46**
46	Postpronotal lobe fuscous or orange-brown anteriorly and yellow posteriorly; abdominal tergites III–V with a broad medial longitudinal black stripe and with or without narrow sublateral longitudinal black bands over tergites III–V which are all joined across posterior margin of tergite V by a narrow transverse black band	**47**
–	Postpronotal lobe entirely yellow; abdominal tergites III–V with either a narrow medial longitudinal black band on all tergites or a broad medial band on tergite V only	**48**
47	Abdomen with broad sublateral black stripes on tergites III–V, in addition to medial stripe (Fig. 13A–E) (cue-lure) (bred from *Medusantheralaxiflora* in Papua New Guinea)	**B. (Bactrocera) enochra (Drew)**
–	Abdomen with sublateral black stripes absent on tergites III–V (Fig. 12A–F) (cue-lure)	**B. (Bactrocera) quasienochra sp. nov.**
48	Anepisternal stripe reaching anterior notopleural seta; bulla present in male wing (Fig. 27A–D) (bred from *Gnetumgnemon*)	**B. (Bulladacus) pacificae Drew & Romig**
–	Anepisternal stripe reaching midway between anterior margin of notopleuron and anterior notopleural seta; no bulla in male wing (Fig. 27E–G) (cue-lure)	**B. (Neozeugodacus) buinensis Drew**
49	Scutum basically red-brown	**50**
–	Scutum predominantly black	**52**
50	Lateral postsutural vitta very short and tapering to a point posteriorly; a circular black spot present on tergite V (Fig. 27H–J)	**B. (Bulladacus) unipunctata (Malloch)**
–	Lateral postsutural vitta long and reaching to intra-alar seta; abdominal tergites uniformly pale colored or with patterns of dark markings	**51**
51	Costal cells colorless; abdominal tergites III–V with broad lateral longitudinal fuscous stripes (Fig. 27K–M)	**B. (Bactrocera) aithogaster Drew**
–	Costal cells with pale fuscous coloration; abdominal tergites III–V uniformly orange-brown or with a black ‘T’-shaped pattern (Fig. 28A–C) (cue-lure, zingerone) (bred from *Inocarpusfagifer*)	**B. (Bactrocera) moluccensis (Perkins)**
52	Postpronotal lobe dark fuscous (Fig. 28D–F) (cue-lure)	**B. (Bactrocera) furvescens Drew**
–	Postpronotal lobe yellow	**53**
53	Abdominal tergites entirely black (Fig. 28G–I) (cue-lure)	**B. (Bactrocera) aterrima (Drew)**
–	Abdominal tergites orange-brown with or without dark color patterns	**54**
54	Abdominal tergites either entirely orange-brown or with very narrow black lines anterolaterally on tergite III and occasionally with a narrow medial black stripe over tergites III–V (Fig. 26G–I) (methyl eugenol) (bred from Moraceae in Australia and *Pimelodendronamboicinum* in Papua New Guinea)	**some specimens of B. (Bactrocera) bancroftii (Tryon)**
–	Abdominal tergites orange-brown with distinct dark markings laterally and medially	**55**
55	Medial postsutural vitta present (Fig. 18E–H) (bred from *Terminalia catappa* and *Gnetumgnemon*)	**females of B. (Bulladacus) penefurva Drew**
–	Medial postsutural vitta absent (Fig. 28J–L) (methyl eugenol)	**B. (Bactrocera) parafroggatti Drew & Romig**

## Discussion

The snap-shot survey yielded 16,843 Dacine flies, belonging to 30 known and six new species, described herein, increasing the number of species known from the Solomon Islands from 48 to 54 (Table 1). Twenty-eight species were represented by at least ten specimens and the five most collected species were *Bactrocerafrauenfeldi* (Schiner) (43.0% of all specimens), *B.froggatti* (Bezzi) (13.4%), *B.umbrosa* (Fabricius) (9.6%), *B.morula* Drew (7.1%), and *B.pagdeni* (Malloch) (7.0%). Our sampling effort was very fruitful, yielding 29 of the 37 species previously collected by trapping over eight years, plus we found six new species, and 18 new island records (Table 1). We collected 31 of 48, 22 of 31, and 8 of 14 species found on Guadalcanal, Kolombangara, and Gizo, respectively. The species accumulation curves (Fig. 2) demonstrate the highest species diversity to be in the forests of Guadalcanal, with twice as many species as in agricultural sites (Fig. 2C). Despite the deployment of 36 sets of traps in the rich protected forests of Kolombangara (688 km^2^), the number of collected and projected species was still half as many as on Guadalcanal (5,302 km^2^) (Fig. 2B, C), consistent with previously published accounts (Drew and Romig 2001; Hollingsworth et al. 2003). The difference is related to island size, with number of fruit fly species clearly correlated (r^2^ = 60.9%) to island size in the Solomon Islands (Suppl. material 3: Fig. S2).

**Figure 18.**
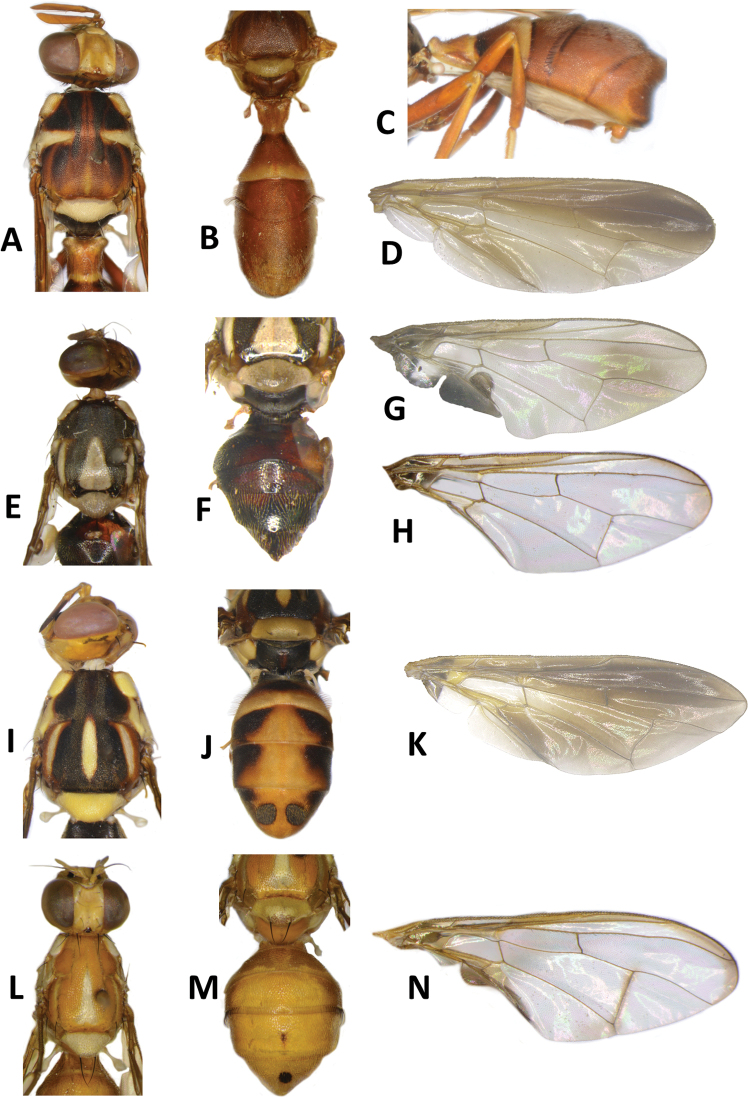
*Dacussolomonensis* Malloch **A** head and scutum **B, C** abdomen **D** wing. *Bactrocerapenefurva* Drew **E** head and scutum **F** abdomen **G** male wing **H** female wing. *Bactrocerapagdeni* (Malloch) **I** head and scutum **J** abdomen **K** wing. *Zeugodacusunivittatus* (Drew) **L** head and scutum **M** abdomen **N** wing.

**Figure 19.**
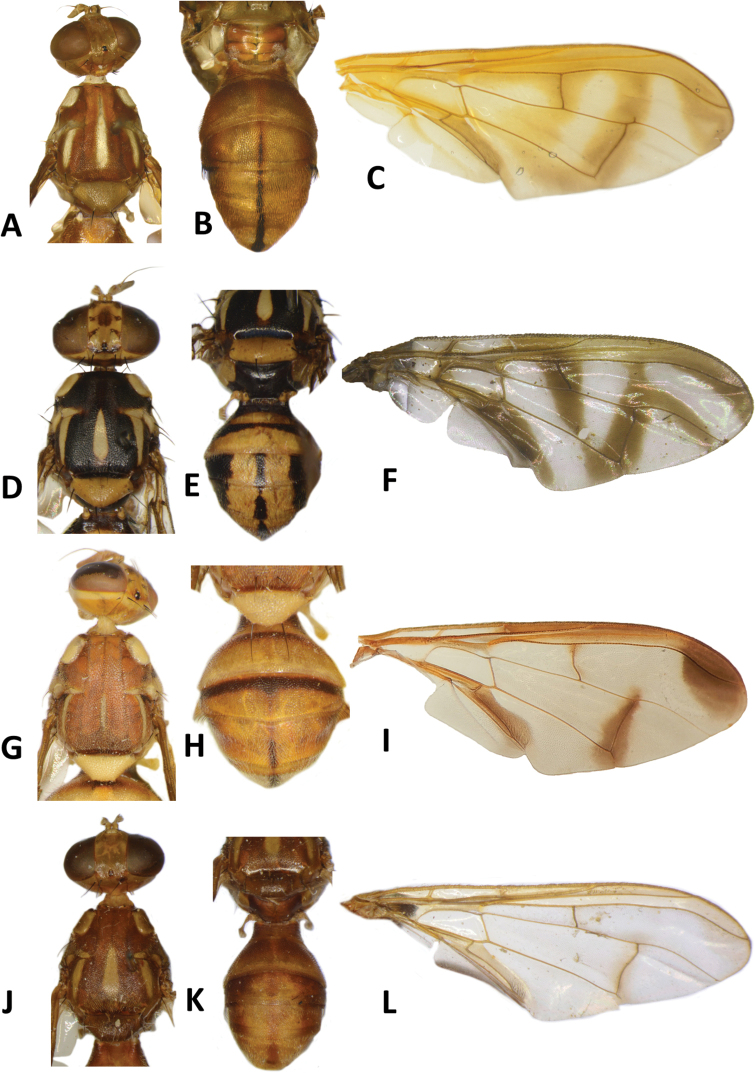
*Zeugodacushamaceki* Drew & Romig **A** head and scutum **B** abdomen **C** wing. *Zeugodacusamoenus* (Drew) **D** head and scutum **E** abdomen **F** wing. *Zeugodacuscucurbitae* (Coquillett) **G** head and scutum **H** abdomen **I** wing. *Zeugodacusfuscipennulus* (Drew & Romig) **G** head and scutum **H** abdomen **I** wing.

**Figure 20.**
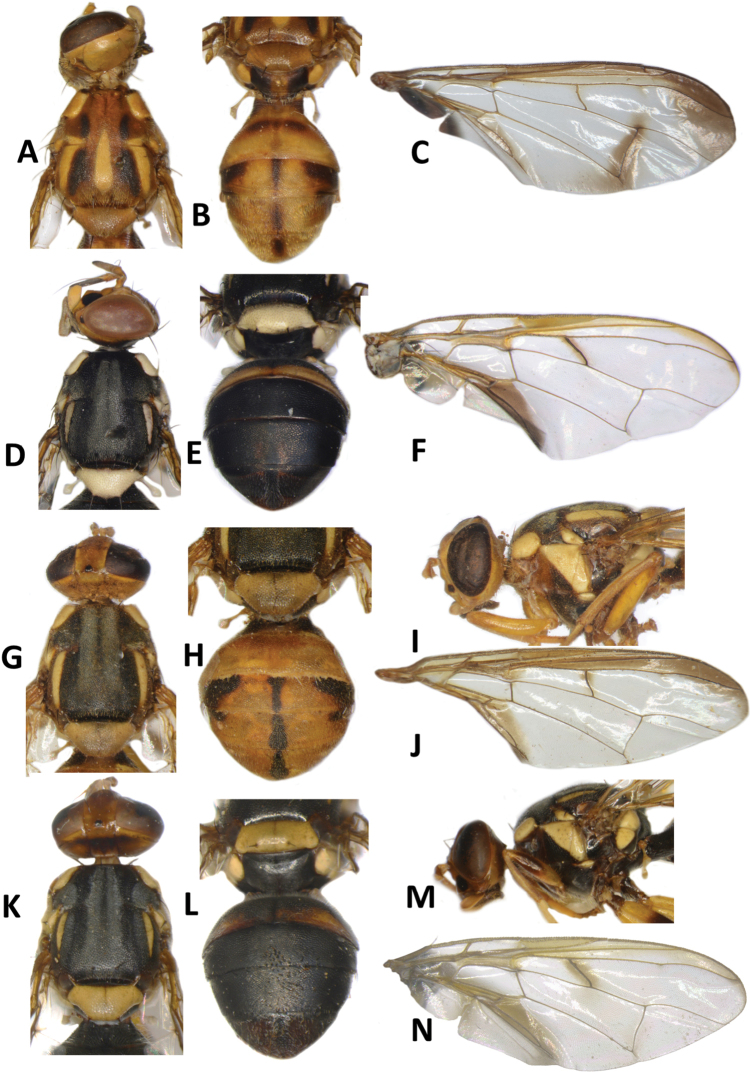
*Zeugodacusabdoangustus* (Drew) **A** head and scutum **B** abdomen **C** wing. *Bactroceramelanogaster* Drew **D** head and scutum **E** abdomen **F** wing. *Bactroceraphaea* (Drew) **G** head and scutum **H** abdomen **I** lateral view **J** wing. *Bactroceraneonigrita* Drew **K** head and scutum **L** abdomen **M** lateral view **N** wing.

**Figure 21.**
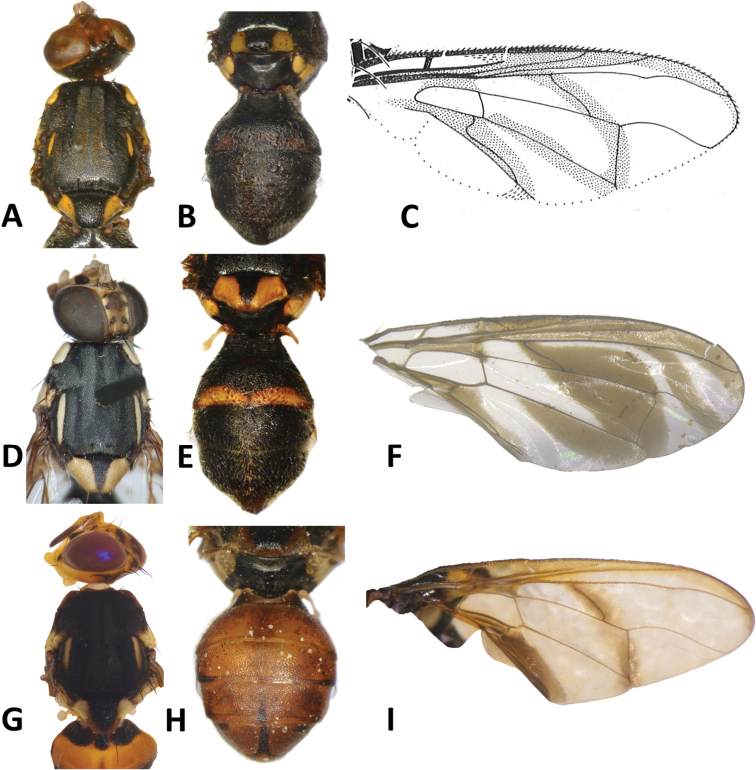
*Bactrocerareclinata* Drew **A** head and scutum **B** abdomen **C** wing (reproduced from Drew 1989). *Bactroceralongicornis* Macquart **D** head and scutum **E** abdomen **F** wing. *Bactrocerahollingsworthi* Drew & Romig **G** head and scutum **H** abdomen **I** wing.

In addition to collecting three new species, the use of zingerone lure revealed that *Bactrocerapagdeni*, formerly known only by its female holotype (Drew 1974), one specimen at the Bishop Museum collections (BPBM), and a few males recently captured in zingerone traps (Hancock and Drew 2018b), is actually a common and widespread species, with 1,174 specimens collected during our survey (Table 1). Likewise, the recent discovery of the attraction of *Bactroceraquadrisetosa* (Bezzi) to dihydroeugenol and isoeugenol lures in Vanuatu (Leblanc, unpublished) will likely reveal that this species is also common and widespread in the Solomon Islands. Clearly, many new species are awaiting discovery with the increasing availability of new generation lures (Manrakhan et al. 2017; Royer et al. 2018, 2019). Several rare species that require further attention in future surveys include: *B.aithogaster* Drew (known by only two specimens), *B.bancroftii* (Tryon) (one specimen of this Australian species from Guadalcanal), *B.furvescens* Drew, a Papua New Guinea species of which a single specimen was collected in 1971 in Honiara, and *B.unipunctata* (Malloch) known from a single specimen collected on Florida Island (Malloch 1939). The species from Mount Austen (Guadalcanal), identified as *B.musae* (Tryon) (Drew and Romig 2001), is likely a non-pest species member of the *B.musae* complex (Drew et al. 2011). *Bactroceramusae* is a major pest of banana, and no fruit fly infestations have been observed on banana in the Solomon Islands, even in recent years (FT, pers. obs.).

**Figure 22.**
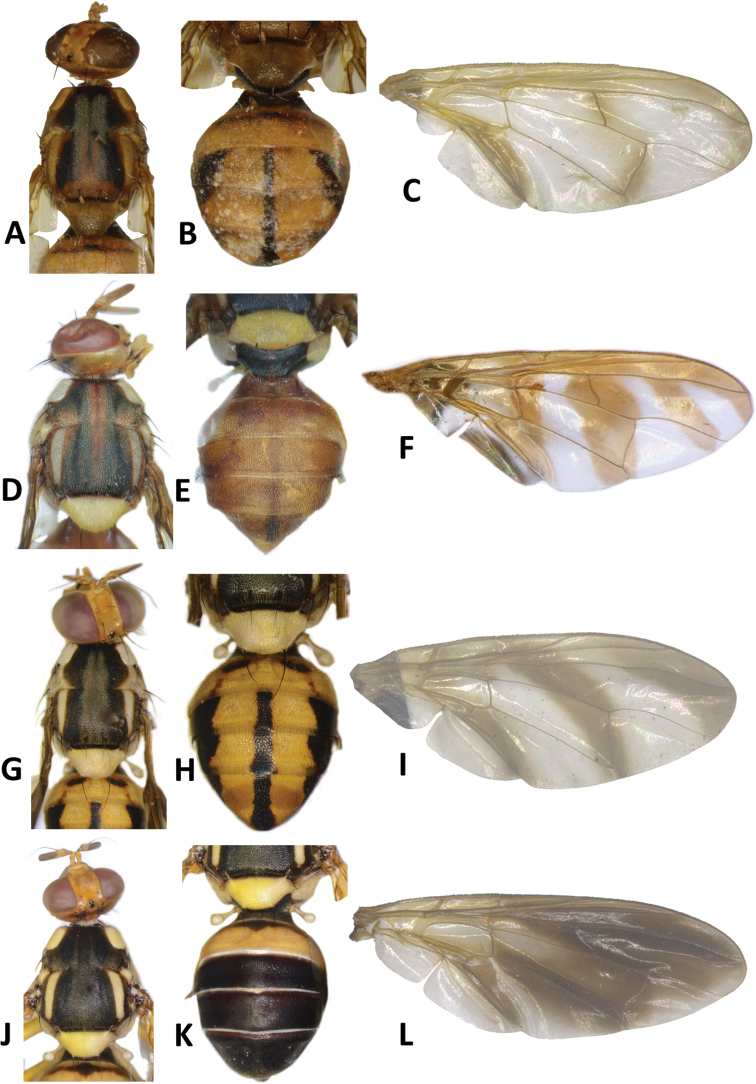
*Bactroceraunitaeniola* Drew & Romig **A** head and scutum **B** abdomen **C** wing. *Bactroceraumbrosa* (Fabricius) **D** head and scutum **E** abdomen **F** wing. *Bactroceraunifasciata* (Malloch) **G** head and scutum **H** abdomen **I** wing. *Bactrocerapepisalae* (Froggatt) **J** head and scutum **K** abdomen **L** wing.

**Figure 23.**
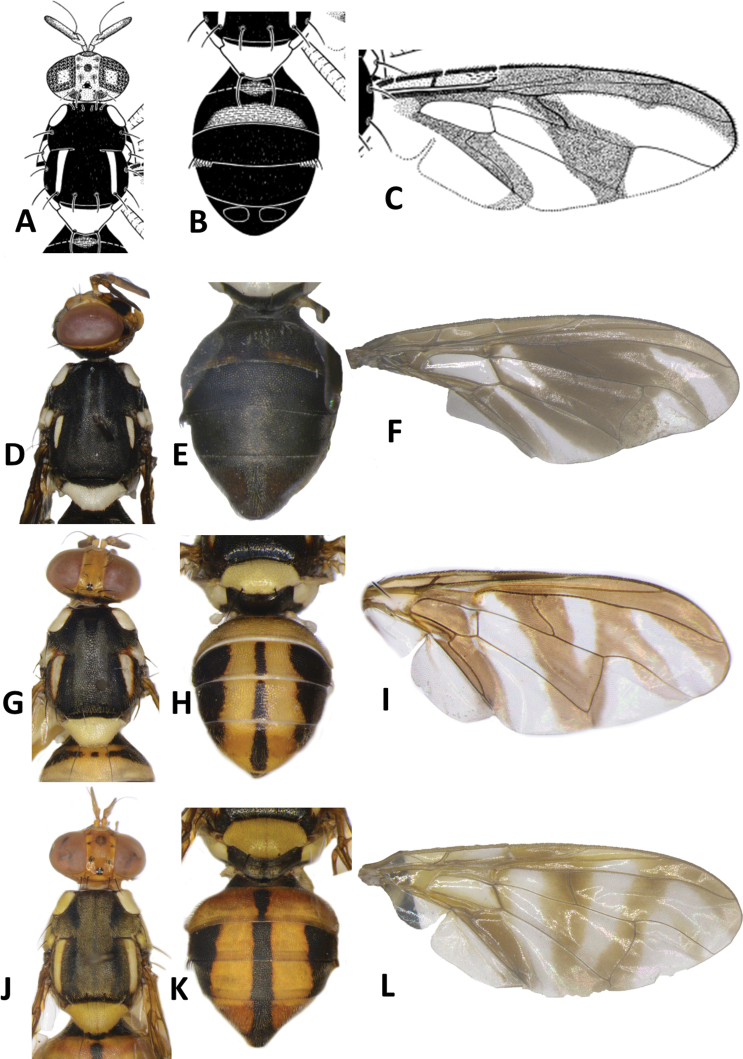
*Bactroceraobliquivenosa* Drew & Romig (reproduced from Drew and Romig 2001) **A** head and scutum **B** abdomen **C** wing. *Bactrocerabiarcuata* (Walker) **D** head and scutum **E** abdomen **F** wing. *Bactroceranigrescentis* (Drew) **G** head and scutum **H** abdomen **I** wing. *Bactroceraredunca* (Drew) **J** head and scutum **K** abdomen **L** wing.

**Figure 24.**
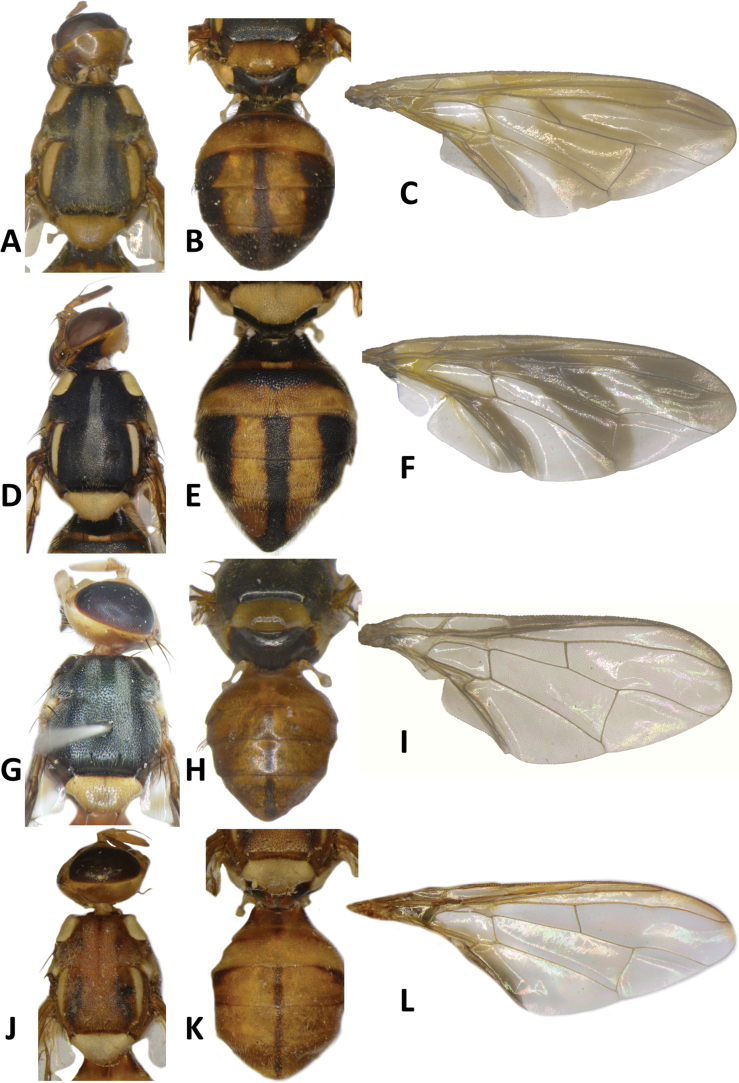
*Bactroceraconfluens* (Drew) **A** head and scutum **B** abdomen **C** wing. *Bactroceradecumana* (Drew) **D** head and scutum **E** abdomen **F** wing. *Bactroceraminuta* (Drew) **G** head and scutum **H** abdomen **I** wing. *Bactroceranaucleae* (Drew & Romig) **J** head and scutum **K** abdomen **L** wing.

The COI sequences we obtained for the new species typically have large minimum pairwise distances to their nearest congeners, up to 12%, whereas the average minimum distance between species for *Bactrocera* is 6.09% (Suppl. material 2: Table S1; Doorenweerd et al. 2020). This is likely due to a lack of species from Papua New Guinea represented in the reference dataset, where there is a large, mostly unstudied, diversity of *Bactrocera* (White and Evenhuis 1999; Drew pers. comm.). As a consequence, the currently available reference data suggests that COI reliably distinguishes all newly described species, but further sampling of species in New Guinea may reduce the pairwise distance resolution (Doorenweerd et al. 2020). There is one potentially new species (*B.* spnSol08; molecular voucher UHIM.ms08767), for which we have one specimen, that we leave undescribed. Although its COI sequence is highly divergent, closest to *B.hantanae* Tsuruta & White at 9.89%, there is only a single specimen and its morphology has no apparent differences with that of *B.dorsalis* (Hendel). Future sampling will hopefully bring in a larger series of this potentially new species to enable further examination of the morphological characters.

**Figure 25.**
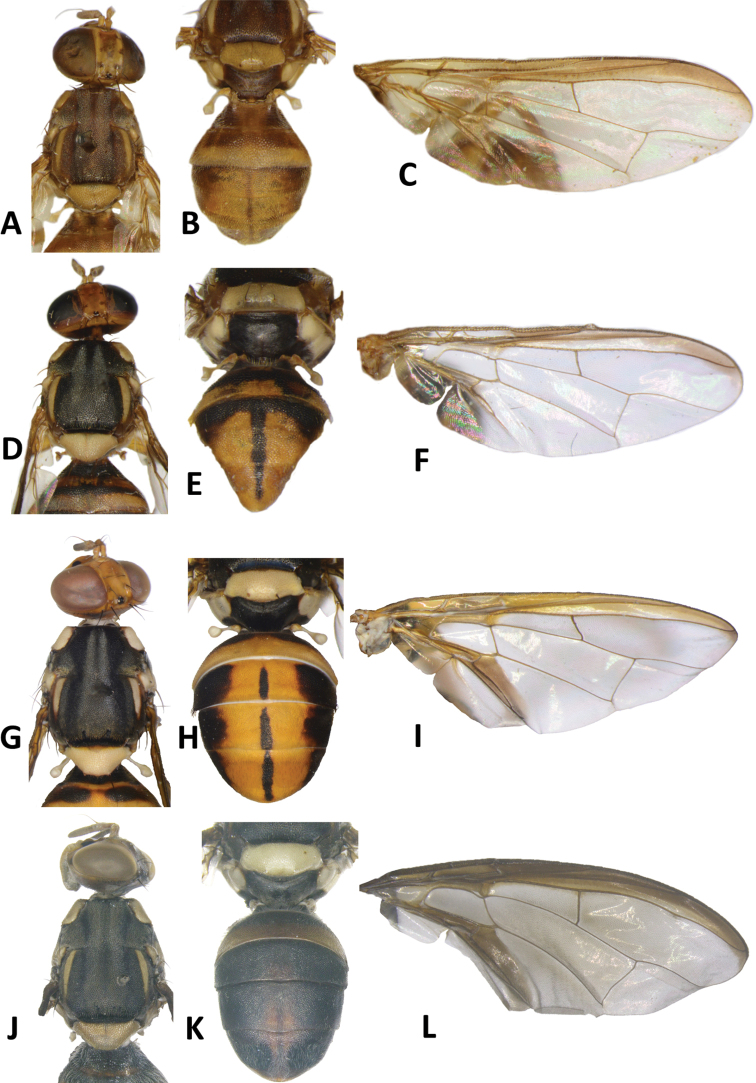
*Bactrocerahastigerina* (Hardy) **A** head and scutum **B** abdomen **C** wing. *Bactroceracalophylli* (Perkins & May) **D** head and scutum **E** abdomen **F** wing. *Bactrocerafroggatti* (Bezzi) **G** head and scutum **H** abdomen **I** wing. *Bactroceraquadrisetosa* (Bezzi) **J** head and scutum **K** abdomen **L** wing.

**Figure 26.**
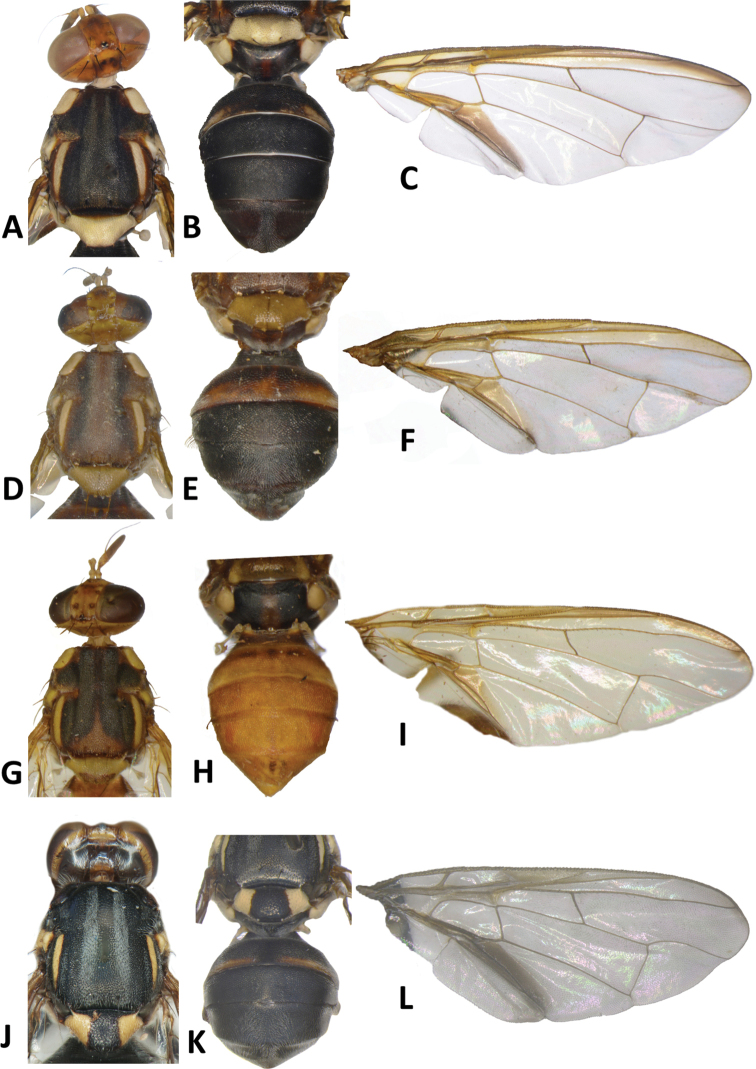
*Bactroceraepicharis* (Hardy) **A** head and scutum **B** abdomen **C** wing. *Bactroceraatrabifasciata* Drew & Romig **D** head and scutum **E** abdomen **F** wing. *Bactrocerabancroftii* (Tryon) (specimen from Australia) **G** head and scutum **H** abdomen **I** wing. *Bactrocerapicea* (Drew) **J** head and scutum **K** abdomen **L** wing.

In addition to the data from this survey, we summarized trapping data in the Solomon Islands generated during the Regional Fruit Fly Projects in the Pacific, as a further indication of the relative abundance and to update the distribution of each species (Table 1). Over 1.8 million flies were collected from 180 sites maintained throughout the archipelago between 1994 and 2001 (Vagalo et al. 1997; Drew and Romig 2001; Hollingsworth et al. 2003; Leblanc et al. 2012). A few specimens of then undescribed *B.geminosimulata* and *B.quasienochra* may have been included among these records.

**Table 1.** Checklist of Dacine fruit flies of Solomon Islands, including number of specimens collected during the Regional Fruit Fly Projects (1994–2001) and the 2018 survey. References to earliest record for each group on islands are: A: Macquart 1835, B: Froggatt 1910, C: Bezzi 1919, D: Malloch 1939, E: Hardy 1954, F: Drew 1972, G: Drew 1974, H: Eta 1985, I: Drew 1989, J: Waterhouse 1993; K: Hollingsworth et. al. 1997, L: Drew and Romig 2001, new: previously unpublished or new record, W: widespread species.

Species	Lure	# trapped Solomon Islands (1994–2001)	# trapped Gizo (2018)	# trapped Kolombangara (2018)	# trapped Guadalcanal (2018)	Shortland Group	Choiseul	Vella Lavella	Gizo	Kolombangara	New Georgia	Isabel	Russell	Florida (Ngella & Savo)	Guadalcanal	Malaita	San Cristobal	Rennell & Bellona	Santa Cruz	Reef Islands
*** BACTROCERA ***																				
*B.aithogaster* Drew, 1989	No known lure														I					
B.*allodistincta* Leblanc & Doorenweerd	Cue-lure				12										new					
*B.aterrima* (Drew, 1972)	Cue-lure	10			1	I	L			L		L			new					
*B.atrabifasciata* Drew & Romig, 2001	Cue-lure	14	1		1	L			L			L		L	new					
*B.bancroftii* (Tryon, 1927)	Methyl eugenol	118													L					
*B.biarcuata* (Walker, 1865)	Methyl eugenol	299		28	3	I	L		L	L	L	L	L	L	L		L			
*B.buinensis* Drew, 1989	Cue-lure	16		163			L			L					new		new			
*B.calophylli* (Perkins & May, 1949)	No known lure	bred from fruit													L					
*B.confluens* (Drew, 1971)	Methyl eugenol	412			1		new			new				L	I	new				
*B.decumana* (Drew, 1972)	Cue-lure	1,226		208		I	L	L	L	L		L	L	L	L	L	L			
*B.enochra* (Drew, 1972)	Cue-lure	33	1	19	1	F			new	L		L			L					
*B.epicharis* (Hardy, 1970)	Cue-lure	168		119	48	I	new			L		new		L	L	L				
*B.frauenfeldi* (Schiner, 1868)	Cue-lure, zingerone	1,271,832	1686	921	4636	W	W	W	W	W	W	W	D	W	W	W	W	W	W	W
*B.froggatti* (Bezzi, 1928)	Methyl eugenol	33,514	36	983	1236	I	L	L	G	L	L	L	C	D	D	L	L	L		
*B.furvescens* Drew, 1989	Cue-lure														I					
*B.geminosimulata* Leblanc & Doorenweerd	Cue-lure				14										new					
*B.hastigerina* (Hardy, 1954)	No known lure	bred from fruit													L					
*B.hollingsworthi* Drew & Romig, 2001	Cue-lure	5		1						new									L	
B.*kolombangarae* Leblanc & Doorenweerd	Zingerone			18	1					new					new					
*B.longicornis* Macquart, 1835	Cue-lure											A								
*B.melanogaster* Drew, 1989	Methyl eugenol	820		2	10	new	I	new	new	I	I	I	I	I	I					
*B.minuta* (Drew, 1971)	Cue-lure	45																	L	
*B.moluccensis* (Perkins, 1939)	Cue-lure	71,499	9	5	435	L	L	L	L	L	L	L	L	L	L	L	L	L	L	
*B.morula* Drew, 1989	Cue-lure	861			1202				L						I		L			
*B.naucleae* Drew & Romig, 2001	Methyl eugenol	130					L					L			L					
*B.neonigrita* Drew, 1989	Methyl eugenol					I														
*B.nigrescentis* (Drew, 1971)	Cue-lure	279	1	32	331		L	L	new	new	new	L		L	L			L	L	
*B.obliquivenosa* Drew & Romig, 2001	Methyl eugenol	1								L										
*B.pacificae* Drew & Romig, 2001	No known lure	bred from fruit													L				L	
*B.pagdeni* (Malloch, 1939)	Zingerone			718	456					new				D	new					
*B.parafroggatti* Drew & Romig, 2001	Methyl eugenol	1,645			85		L			new	L	L		L	L		L	L		
*B.penefurva* Drew, 1989	No known lure	bred from fruit													I					
*B.pepisalae* (Froggatt, 1910)	Methyl eugenol	7,746			35	new	L	E		L	L	L	B	D	G	L	G			
*B.phaea* (Drew, 1971)	Cue-lure	97										L		L	L					
*B.picea* (Drew, 1972)	Methyl eugenol	726		227	19	I	L	L	L	L	L	L	I	L	L	new				
*B.pseudodistincta* (Drew, 1971)	Cue-lure	433		5			L			L		L		L	L	L	L	L	L	
*B.quadrisetosa* (Bezzi, 1928)	Dihydroeugenol, isoeugenol	bred from fruit									L			D	I				L	
B.*quasienochra* Leblanc & Doorenweerd	Cue-lure				1										new					
*B.reclinata* Drew, 1989	Methyl eugenol	1													L					
*B.redunca* (Drew, 1971)	Cue-lure	7,031			524	I	L			L		L	L	L	L	L	new		L	
*B.simulata* (Malloch, 1939)	Cue-lure	32,810		16	350	I	L	L	L	L	I	L	L	D	I	L	L	L	L	
B.*tsatsiae* Leblanc & Doorenweerd	Zingerone			20	9					new					new					
*B.umbrosa* (Fabricius, 1805)	Methyl eugenol	362,783	157	170	1296	new	W	W	W	W	W	W	W	D	W	W	W	W	D	W
*B.unifasciata* (Malloch, 1939)	Cue-lure	5		1	18					new		L			D					
*B.unipunctata* (Malloch, 1939)	No known lure													D						
*B.unitaeniola* Drew & Romig, 2001	Cue-lure	13					L					L			L					
B.*vargasi* Leblanc & Doorenweerd	Zingerone			9	34					new					new					
*** DACUS ***																				
*D.solomonensis* Malloch, 1939	Cue-lure	23,085			60		L			new	L	L	L	L	D	L	L			
*** ZEUGODACUS ***																				
*Z.abdoangustus* (Drew, 1972)	Cue-lure	38			11							L			L	L				
*Z.amoenus* (Drew, 1972)	Cue-lure	3										L								
*Z.cucurbitae* (Coquillett, 1899)	Cue-lure, zingerone	43,294	7	305	44	H	J	J	J	J	J	J	K		K	K				
*Z.fuscipennulus* (Drew & Romig, 2001)	Cue-lure	101		1						L	new	new			L		new			
*Z.hamaceki* (Drew & Romig, 2001)	Cue-lure	115			5					L		L			L					
*Z.univittatus* (Drew, 1972)	Cue-lure	118			95					new		L			L		L			

**Figure 27.**
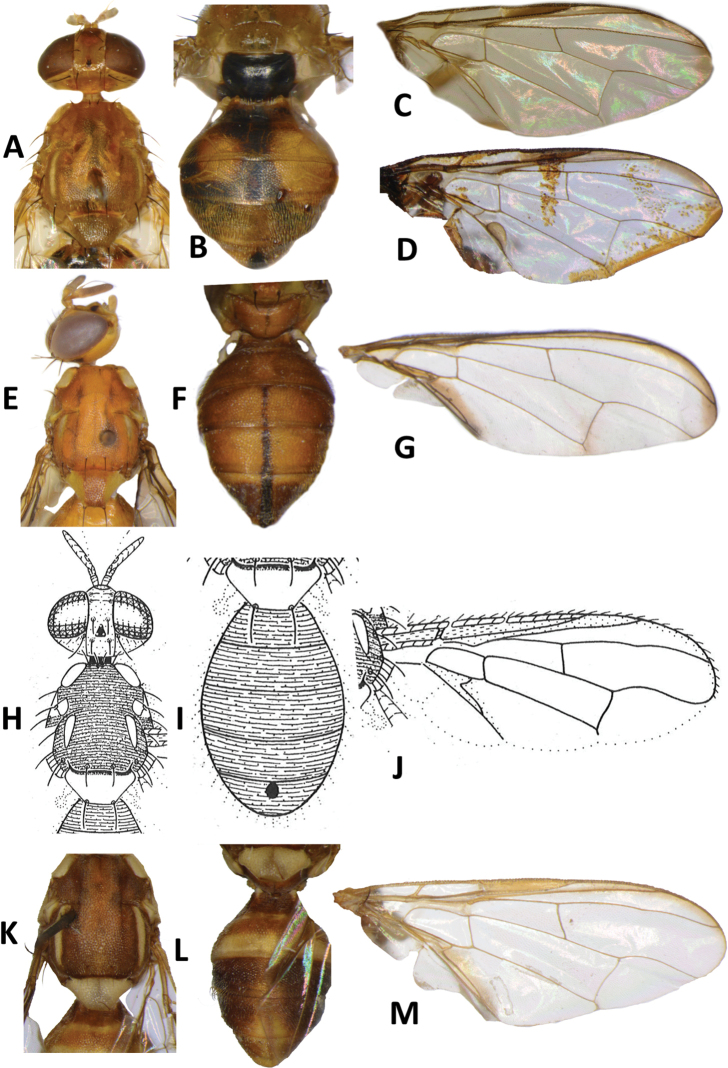
*Bactrocerapacificae* Drew & Romig **A** head and scutum **B** abdomen **C** female wing **D** male wing. *Bactrocerabuinensis* Drew **E** head and scutum **F** abdomen **G** wing. *Bactroceraunipunctata* (Malloch) (reproduced from Drew 1989) **H** head and scutum **I** abdomen **J** wing. *Bactroceraaithogaster* Drew **K** scutum **L** abdomen **M** wing.

**Figure 28.**
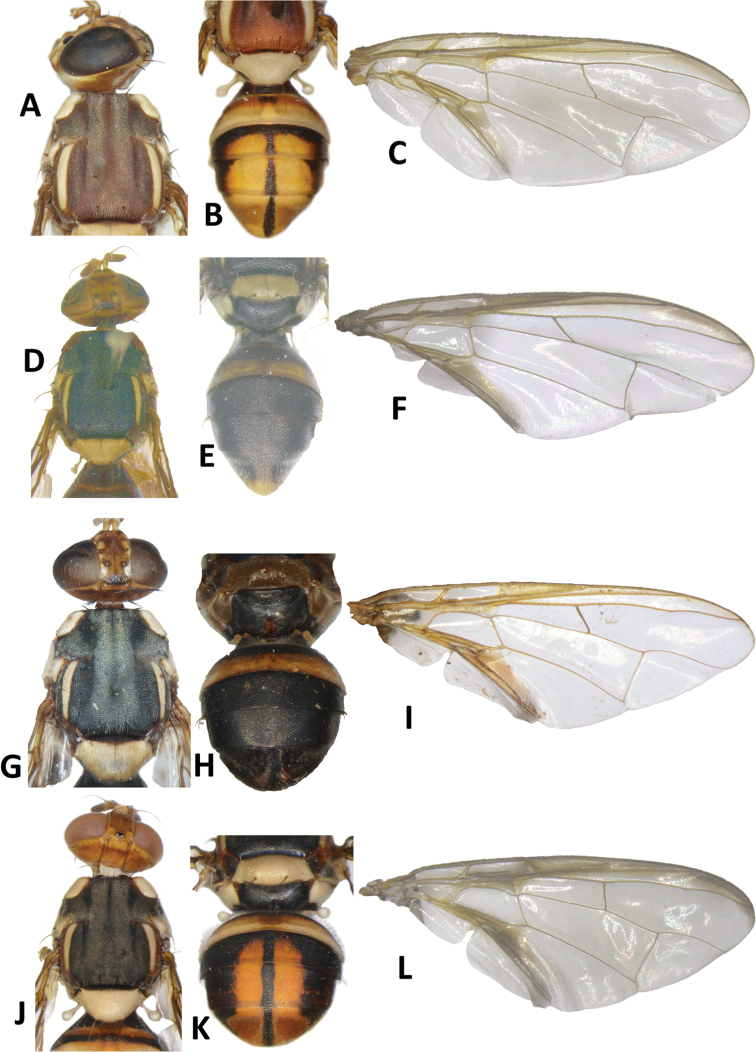
*Bactroceramoluccensis* (Perkins) **A** head and scutum **B** abdomen **C** wing. *Bactrocerafurvescens* Drew **D** head and scutum **E** abdomen **F** wing. *Bactroceraaterrima* (Drew) **G** head and scutum **H** abdomen **I** wing. *Bactroceraparafroggatti* Drew & Romig **J** head and scutum **K** abdomen **L** wing.

## Supplementary Material

XML Treatment for Bactrocera (Bactrocera) allodistincta

XML Treatment for Bactrocera (Bactrocera) geminosimulata

XML Treatment for Bactrocera (Parazeugodacus) kolombangarae

XML Treatment for Bactrocera (Bactrocera) quasienochra

XML Treatment for Bactrocera (Bactrocera) tsatsiai

XML Treatment for Bactrocera (Bactrocera) vargasi

## Supplementary materials

Supplementary material 1 Figure S1. COI Phylogeny Data type phylogenetic Explanation note Maximum likelihood tree based on COI sequence data, modified from Doorenweerd et al. (2020) to have species / species complexes ‘collapsed’ into triangles where the horizontal width indicates the maximum pairwise distance of that clade. Newly described species are indicated in green. https://binary.pensoft.net/file/581791This dataset is made available under the Open Database License (http://opendatacommons.org/licenses/odbl/1.0/). The Open Database License (ODbL) is a license agreement intended to allow users to freely share, modify, and use this Dataset while maintaining this same freedom for others, provided that the original source and author(s) are credited.Camiel Doorenweerd

Supplementary material 2 Table S1. Pairwise molecular distance Data type phylogenetic Explanation note COI Pairwise distance statistics for the *Bactrocera* included in Doorenweerd et al. (2020), including the species newly described here. https://binary.pensoft.net/file/581792This dataset is made available under the Open Database License (http://opendatacommons.org/licenses/odbl/1.0/). The Open Database License (ODbL) is a license agreement intended to allow users to freely share, modify, and use this Dataset while maintaining this same freedom for others, provided that the original source and author(s) are credited.Camiel Doorenweerd

Supplementary material 3 Figure S2. Number of dacine fruit fly species in relation to island size in the Solomon Islands Data type pdf. file https://binary.pensoft.net/file/581793This dataset is made available under the Open Database License (http://opendatacommons.org/licenses/odbl/1.0/). The Open Database License (ODbL) is a license agreement intended to allow users to freely share, modify, and use this Dataset while maintaining this same freedom for others, provided that the original source and author(s) are credited.Luc Leblanc, Francis Tsatsia, Camiel Doorenweerd
