# Supplementary figures and images for: Novel lures and COI sequences reveal cryptic new species of Bactrocera fruit flies in the Solomon Islands (Diptera, Tephritidae, Dacini)
Source: Zookeys. 2021 Aug 27;1057:49–103. doi: 10.3897/zookeys.1057.68375 (PMC8417025; doi:10.3897/zookeys.1057.68375)

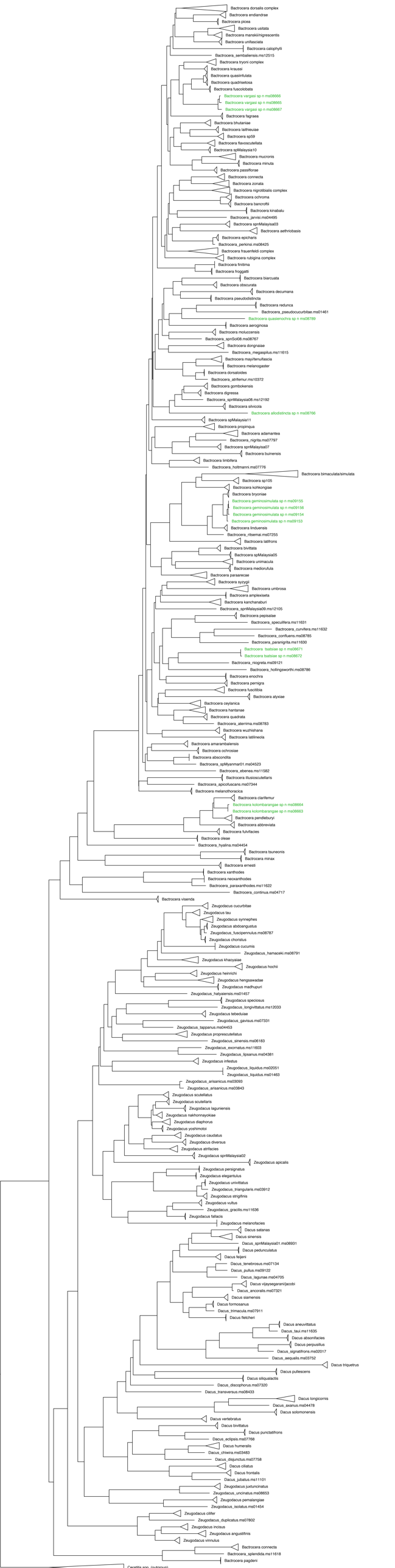

Supplement: Supplementary material 1 — Figure S1. COI Phylogeny [file zookeys-1057-049-s001.pdf]
