# Supplementary material for: Novel lures and COI sequences reveal cryptic new species of Bactrocera fruit flies in the Solomon Islands (Diptera, Tephritidae, Dacini)
Source: Zookeys. 2021 Aug 27;1057:49–103. doi: 10.3897/zookeys.1057.68375 (PMC8417025; doi:10.3897/zookeys.1057.68375)
Supplement: Supplementary material 2 — Table S1. Pairwise molecular distance [file zookeys-1057-049-s002.pdf]

species	avg_intra	intra_d_max	n_intra	avg_inter	inter_dmin_nn	n_inter	nearest_neighbor
Bactrocera abbreviata	0.00428621	0.008042895	153	0.144668553	0.003351206	56772	ms07333.Bactrocera_bipustulata
Bactrocera abscondita	N/A	N/A	0	0.101805471	0.051574012	3171	ms04523.Bactrocera_spMyanmar01
Bactrocera adamantea	0.02411253	0.034159411	21	0.116780271	0.077695914	22155	ms11948.Bactrocera_spnMalaysia07
Bactrocera aeroginosa	0.00066979	0.000669792	1	0.116310304	0.102546917	6340	ms08775.Bactrocera_moluccensis
Bactrocera aethriobasis	0.02076356	0.020763563	1	0.129362963	0.111855325	6340	ms03741.Bactrocera_zonata
Bactrocera albistrigata	0.01769861	0.032819826	253	0.086148209	0.000669792	72427	ms11142.Bactrocera_caledoniensis
Bactrocera allodistincta sp n	N/A	N/A	0	0.128379273	0.102546917	3171	ms03573.Bactrocera_gombokensis
Bactrocera alyxiae	0.00446528	0.004688547	3	0.118890172	0.103817816	9507	ms05926.Bactrocera_zonata
Bactrocera amarambalensis	0.00589417	0.008707301	15	0.099402751	0.079035499	18996	ms04553.Bactrocera_abscondita
Bactrocera amplexiseta	0.00066979	0.000669792	1	0.105055819	0.091823056	6340	ms09118.Bactrocera_syzygii
Bactrocera apicofuscans	N/A	N/A	0	0.105131055	0.093101139	3171	ms10293.Bactrocera_carambolae
Bactrocera aquilonis	0.00707663	0.013404826	120	0.090126222	0.001339585	50496	ms01517.Bactrocera_tryoni
Bactrocera aterrima	N/A	N/A	0	0.09879156	0.063672922	3171	ms04539.Bactrocera_quadrata
Bactrocera atrifemur	N/A	N/A	0	0.103147652	0.030810449	3171	ms04389.Bactrocera_dorsaloides
Bactrocera bancroftii	0.0031257	0.004688547	3	0.097252421	0.023442733	9507	ms10489.Bactrocera_ochroma
Bactrocera bhutaniae	0.00601139	0.014354067	820	0.083583079	0.05870021	128371	ms11148.Bactrocera_mucronis
Bactrocera biarcuata	0.00089351	0.001340483	3	0.111342279	0.090421969	9507	ms11642.Bactrocera_obscurata
Bactrocera bimaculata	0.00600956	0.010723861	91	0.132356446	0.063672922	44212	ms08675.Bactrocera_simulata
Bactrocera bipustulata	N/A	N/A	0	0.144110692	0.003351206	3171	ms12048.Bactrocera_abbreviata
Bactrocera bivittata	0.00293608	0.006702413	55	0.111343951	0.065013405	34771	ms08978.Bactrocera_spMalaysia05
Bactrocera bryoniae	0.00178701	0.003351206	6	0.110534431	0.014745308	12672	ms09154.Bactrocera_spSol12
Bactrocera buinensis	0.00167448	0.002679169	6	0.119465515	0.102546917	12672	ms07797.Bactrocera_nigrita
Bactrocera cacuminata	0.01054199	0.021433356	45	0.059649652	0.010046885	31620	ms08784.Bactrocera_parafroggatti
Bactrocera caledoniensis	0.02042867	0.035498995	6	0.086741542	0.005358339	12672	ms08820.Bactrocera_albistrigata
Bactrocera calophylli	N/A	N/A	0	0.11305941	0.098459478	3171	ms10644.Bactrocera_raiensis
Bactrocera carambolae	0.00950828	0.018096515	3655	0.050150534	0	265396	ms10194.Bactrocera_raiensis
Bactrocera ceylanica	0.012958	0.024798928	6	0.098869459	0.081044876	12672	ms03769.Bactrocera_hantanae
Bactrocera clarifemur	0.00489251	0.009383378	136	0.147315618	0.040187542	53635	ms12057.Bactrocera_pendleburyi
Bactrocera commensurata	0.00545504	0.013708019	171	0.057084814	0.016075017	59907	ms12139.Bactrocera_occipitalis
Bactrocera confluens	N/A	N/A	0	0.124692535	0.103286385	3171	ms11632.Bactrocera_curvifera
Bactrocera connecta	0.0154244	0.02147651	6	0.171463823	0.08506363	12672	ms11618.Bactrocera_splendida
Bactrocera continua	N/A	N/A	0	0.160536225	0.147197839	3171	ms08964.Bactrocera_nigrotibialis
Bactrocera correcta	0.00840557	0.016075017	4753	0.089828764	0.055630027	301252	ms05993.Bactrocera_zonata
Bactrocera curvifera	N/A	N/A	0	0.135244505	0.103286385	3171	ms08785.Bactrocera_confluens
Bactrocera curvipennis	N/A	N/A	0	0.089766679	0.01406564	3171	ms01497.Bactrocera_tryoni
Bactrocera decumana	0.00335121	0.003351206	1	0.119165346	0.097855228	6340	ms08584.Bactrocera_pseudodistincta
Bactrocera digressa	0.00680956	0.009377093	6	0.100728489	0.047555258	12672	ms03573.Bactrocera_gombokensis
Bactrocera dongnaiae	0.00725424	0.014745308	136	0.112809702	0.094504021	53635	ms01294.Bactrocera_paraarecae
Bactrocera dorsalis	0.01032319	0.076407507	732655	0.07176343	0	2374771	ms02018.Bactrocera_carambolae
Bactrocera dorsaloides	0.00066979	0.000669792	1	0.098158832	0.030810449	6340	ms10372.Bactrocera_atrifemur
Bactrocera ebenea	N/A	N/A	0	0.120727119	0.103217158	3171	ms09083.Bactrocera_hantanae
Bactrocera endiandrae	0.00835414	0.014735432	55	0.089720306	0.066353887	34771	ms08612.Bactrocera_picea
Bactrocera enochra	0.00134003	0.002010724	6	0.11627171	0.103817816	12672	ms01301.Bactrocera_kanchanaburi
Bactrocera epicharis	0.00312779	0.004021448	3	0.100588974	0.010053619	9507	ms08425.Bactrocera_perkinsi
Bactrocera ernesti	0.00341297	0.003412969	1	0.148314526	0.131367292	6340	ms05993.Bactrocera_zonata
Bactrocera fagraea	0.00281313	0.004018754	10	0.101047463	0.081044876	15835	ms04561.Bactrocera_kraussi
Bactrocera finitima	N/A	N/A	0	0.09420353	0.048927614	3171	ms08607.Bactrocera_froggatti
Bactrocera flavoscutellata	0.00610514	0.012056263	45	0.091044306	0.065683646	31620	ms12193.Bactrocera_spMalaysia10
Bactrocera frauenfeldi	0.00963903	0.018096515	91	0.084748743	0.000669792	44212	ms08445.Bactrocera_albistrigata
Bactrocera froggatti	0.00201072	0.002010724	1	0.09637942	0.048927614	6340	ms11628.Bactrocera_finitima
Bactrocera fulvifacies	0.00312929	0.004024145	3	0.144578707	0.075067024	9507	ms08663.Bactrocera_spnSol06
Bactrocera fuscitibia	0.01057671	0.019138756	66	0.109211806	0.089752177	37920	ms01797.Bactrocera_hantanae
Bactrocera fuscolobata	0.00268097	0.002680965	1	0.087654422	0.044265594	6340	ms12004.Bactrocera_quadrisetosa
Bactrocera geminosimulata sp n	0.00379803	0.00536193	6	0.110926033	0.014745308	12672	ms04500.Bactrocera_bryoniae
Bactrocera gombokensis	0.00446925	0.007372654	21	0.09527491	0.047555258	22155	ms04673.Bactrocera_digressa
Bactrocera hantanae	0.01471185	0.039544236	66	0.098290762	0.062960482	37920	ms04542.Bactrocera_quadrata
Bactrocera hollingsworthi	N/A	N/A	0	0.138282464	0.118632708	3171	ms03769.Bactrocera_hantanae
Bactrocera holtmanni	N/A	N/A	0	0.113660379	0.075737265	3171	ms12188.Bactrocera_limbifera
Bactrocera hyalina	N/A	N/A	0	0.143802628	0.130609511	3171	ms12008.Bactrocera_kirki
Bactrocera illusioscutellaris	0.00312689	0.004018754	3	0.115840051	0.104557641	9507	ms10034.Bactrocera_dorsalis
Bactrocera jarvisi	N/A	N/A	0	0.104648693	0.088152327	3171	ms11590.Bactrocera_psidii
Bactrocera kanchanaburi	0.0087213	0.025469169	190	0.098862006	0.083948959	63040	ms11877.Bactrocera_spBali01
Bactrocera kandiensis	0.01417579	0.073726542	3403	0.07972956	0	256387	ms10959.Bactrocera_dorsalis
Bactrocera kinabalu	0.00100506	0.001340483	6	0.118332702	0.095844504	12672	ms00894.Bactrocera_kirki
Bactrocera kirki	0.00415271	0.006697924	15	0.082255613	0.030140656	18996	ms11136.Bactrocera_psidii
Bactrocera kohkongiae	0.00608806	0.015405224	190	0.113144402	0.077026122	63040	ms01156.Bactrocera_sp105
Bactrocera kolombangarae sp n	0.00201072	0.002010724	1	0.144767798	0.036193029	6340	ms12057.Bactrocera_pendleburyi
Bactrocera kraussi	0.00311789	0.005358339	55	0.084835473	0.056932351	34771	ms04508.Bactrocera_neohumeralis
Bactrocera laithieuiae	0.00513642	0.008037508	6	0.093482144	0.064969859	12672	ms01164.Bactrocera_sp59
Bactrocera lateritaenia	0.00856638	0.014075067	171	0.062623403	0.022103148	59907	ms03540.Bactrocera_thailandica
Bactrocera latifrons	0.00445867	0.010723861	55	0.129855369	0.10924933	34771	ms07255.Bactrocera_ritsemai
Bactrocera latilineola	0.00457691	0.006697924	6	0.107477574	0.094440723	12672	ms11778.Bactrocera_raiensis
Bactrocera limbifera	0.00855862	0.013404826	55	0.103891528	0.075737265	34771	ms07776.Bactrocera_holtmanni

Bactrocera linduensis	0.00536193	0.008042895	3	0.112821078	0.038203753	9507	ms09154.Bactrocera_spSol12
Bactrocera manskii	0.00759098	0.010716678	3	0.086015767	0.014745308	9507	ms08579.Bactrocera_nigrescentis
Bactrocera mayi	0.00773982	0.013395847	36	0.106425603	0.006702413	28467	ms07732.Bactrocera_tenuifascia
Bactrocera mediorufula	0.00200998	0.002679169	6	0.118917107	0.093101139	12672	ms01295.Bactrocera_paraarecae
Bactrocera megaspilus	N/A	N/A	0	0.115185298	0.09919571	3171	ms08767.Bactrocera_spnSol08
Bactrocera melanogaster	0.00067024	0.000670241	1	0.115945497	0.056970509	6340	ms07728.Bactrocera_tenuifascia
Bactrocera melanothoracica	0.00230083	0.003462604	3	0.105264184	0.089082384	9507	ms04470.Bactrocera_frauenfeldi
Bactrocera melas	0.00468855	0.004688547	1	0.08723167	0.004018754	6340	ms04504.Bactrocera_tryoni
Bactrocera minax	0.00223354	0.002679169	3	0.177383015	0.099799062	9507	ms04754.Bactrocera_tsuneonis
Bactrocera minuta	0.00267917	0.002679169	1	0.110895374	0.0770819	6340	ms11150.Bactrocera_mucronis
Bactrocera moluccensis	0.00368633	0.006032172	28	0.110192178	0.085790885	25312	ms08767.Bactrocera_spnSol08
Bactrocera morula	N/A	N/A	0	0.09338884	0.026809651	3171	ms03454.Bactrocera_nigrotibialis
Bactrocera mucronis	0.01555053	0.041928721	15	0.103512112	0.05870021	18996	ms01169.Bactrocera_bhutaniae
Bactrocera musae	0.00390712	0.007367716	36	0.061697094	0.02277294	28467	ms11843.Bactrocera_spBali01
Bactrocera neohumeralis	0.01298653	0.020093771	36	0.087530152	0	28467	ms01497.Bactrocera_tryoni
Bactrocera neoxanthodes	N/A	N/A	0	0.151987505	0.039517749	3171	ms11622.Bactrocera_paraxanthodes
Bactrocera nigrescentis	N/A	N/A	0	0.08722358	0.014745308	3171	ms04491.Bactrocera_manskii
Bactrocera nigrifacia	0.01290021	0.021447721	120	0.097220624	0.002680965	50496	ms07296.Bactrocera_nigrofemoralis
Bactrocera nigrita	N/A	N/A	0	0.115195451	0.075737265	3171	ms11948.Bactrocera_spnMalaysia07
Bactrocera nigrofemoralis	0.0178731	0.024798928	3	0.097208935	0.002680965	9507	ms03813.Bactrocera_nigrifacia
Bactrocera nigrotibialis	0.01442419	0.027461487	378	0.097872958	0.006028131	88032	ms07774.Bactrocera_nigrofemoralis
Bactrocera niogreta	N/A	N/A	0	0.118050806	0.104557641	3171	ms07300.Bactrocera_ceylanica
Bactrocera obscurata	0.00361688	0.006697924	10	0.101166413	0.078418231	15835	ms08867.Bactrocera_propinqua
Bactrocera occipitalis	0.01078683	0.031522468	6670	0.055915827	0	354496	ms05498.Bactrocera_dorsalis
Bactrocera ochroma	0.00959294	0.018084394	190	0.099895717	0.023442733	63040	ms04622.Bactrocera_bancroftii
Bactrocera ochrosiae	0.00204127	0.004018754	21	0.10248115	0.087073007	22155	ms03766.Bactrocera_amarambalensis
Bactrocera oleae	0.00115815	0.002089136	6	0.144268106	0.128600134	12672	ms01295.Bactrocera_paraarecae
Bactrocera OTU5353	0.00941052	0.020763563	6555	0.04998574	0	351555	ms11734.Bactrocera_dorsalis
Bactrocera pagdeni	0.00089366	0.001340483	3	0.167693603	0.115281501	9507	ms11618.Bactrocera_splendida
Bactrocera pallida	0.00169717	0.002679169	15	0.067928211	0.016756032	18996	ms08448.Bactrocera_commensurata
Bactrocera paraarecae	0.01845096	0.034182306	78	0.101163178	0.086461126	41067	ms07300.Bactrocera_ceylanica
Bactrocera parafroggatti	N/A	N/A	0	0.059993828	0.010046885	3171	ms04630.Bactrocera_cacuminata
Bactrocera paranigrita	N/A	N/A	0	0.139332714	0.109993293	3171	ms08785.Bactrocera_confluens
Bactrocera paraxanthodes	N/A	N/A	0	0.147622492	0.039517749	3171	ms12005.Bactrocera_neoxanthodes
Bactrocera passiflorae	0.00602813	0.006028131	1	0.09771599	0.07199424	6340	ms11146.Bactrocera_mucronis
Bactrocera pendleburyi	0.00840839	0.014075067	55	0.14684167	0.036193029	34771	ms08663.Bactrocera_spnSol06
Bactrocera pepisalae	0.0020105	0.003351206	6	0.126773548	0.105898123	12672	ms11631.Bactrocera_speculifera
Bactrocera perigrapha	0.00446828	0.006032172	3	0.083181611	0.024128686	9507	ms10937.Bactrocera_dorsalis
Bactrocera perkinsi	N/A	N/A	0	0.096283315	0.010053619	3171	ms08657.Bactrocera_epicharis
Bactrocera pernigra	0.00737265	0.007372654	1	0.119833023	0.104557641	6340	ms07149.Bactrocera_paraarecae
Bactrocera picea	0.00335121	0.003351206	1	0.090461408	0.066353887	6340	ms04628.Bactrocera_endiandrae
Bactrocera propinqua	0.01006312	0.019423979	990	0.092795688	0.073328541	140715	ms01250.Bactrocera_dorsalis
Bactrocera pseudocucurbitae	N/A	N/A	0	0.137330051	0.117962466	3171	ms08601.Bactrocera_redunca
Bactrocera pseudodistincta	0.00201072	0.002010724	1	0.100363101	0.078418231	6340	ms12010.Bactrocera_obscurata
Bactrocera psidii	0.00867541	0.012056263	21	0.080615652	0.022103148	22155	ms11144.Bactrocera_trilineola
Bactrocera quadrata	0.00535834	0.007367716	3	0.09794425	0.062960482	9507	ms08783.Bactrocera_aterrima
Bactrocera quadrisetosa	0.00402414	0.004024145	1	0.085231777	0.010731053	6340	ms03455.Bactrocera_quasiinfulata
Bactrocera quasienochra sp n	N/A	N/A	0	0.128634574	0.111930295	3171	ms12150.Bactrocera_spMalaysia11
Bactrocera quasiinfulata	0.00602813	0.006028131	1	0.085973513	0.010731053	6340	ms12004.Bactrocera_quadrisetosa
Bactrocera raiensis	0.00795268	0.04822505	7626	0.049680721	0	377952	ms01272.Bactrocera_dorsalis
Bactrocera redunca	0.00134033	0.002010724	3	0.134158437	0.115874079	9507	ms03769.Bactrocera_hantanae
Bactrocera ritsemai	N/A	N/A	0	0.111508293	0.047587131	3171	ms12211.Bactrocera_linduensis
Bactrocera rubigina	0.00546259	0.016085791	3081	0.096978391	0.081714668	244347	ms06083.Bactrocera_zonata
Bactrocera selenophora	N/A	N/A	0	0.070055614	0.036863271	3171	ms10318.Bactrocera_carambolae
Bactrocera sembaliensis	N/A	N/A	0	0.091565603	0.076607387	3171	ms08579.Bactrocera_nigrescentis
Bactrocera silvicola	0.00602813	0.006028131	1	0.114704071	0.083054253	6340	ms04675.Bactrocera_digressa
Bactrocera simulata	0.00589812	0.009383378	15	0.123098279	0.063672922	18996	ms01134.Bactrocera_bimaculata
Bactrocera sp105	0.00616399	0.010723861	55	0.127578104	0.077026122	34771	ms01145.Bactrocera_kohkongiae
Bactrocera sp59	0.0080408	0.011394102	3	0.091732358	0.064969859	9507	ms05345.Bactrocera_laithieuiae
Bactrocera spBali01	0.0070998	0.01138647	10	0.052387606	0.009377093	15835	ms09494.Bactrocera_dorsalis
Bactrocera speculifera	N/A	N/A	0	0.138128392	0.105898123	3171	ms08590.Bactrocera_pepisalae
Bactrocera splendida	N/A	N/A	0	0.168951618	0.08506363	3171	ms06239.Bactrocera_connecta
Bactrocera spMalaysia04	N/A	N/A	0	0.072737836	0.03683858	3171	ms11877.Bactrocera_spBali01
Bactrocera spMalaysia05	0.00401875	0.004018754	1	0.121485995	0.065013405	6340	ms06280.Bactrocera_bivittata
Bactrocera spMalaysia10	0.00401875	0.004018754	1	0.086237486	0.065683646	6340	ms06246.Bactrocera_flavoscutellata
Bactrocera spMalaysia11	0.00603217	0.006032172	1	0.108795267	0.080375084	6340	ms07978.Bactrocera_gombokensis
Bactrocera spMalaysia14	0.00440917	0.010046885	378	0.057590881	0.016744809	88032	ms07629.Bactrocera_carambolae
Bactrocera spMyanmar01	N/A	N/A	0	0.095616604	0.049564635	3171	ms03791.Bactrocera_zonata
Bactrocera spnMalaysia03	0.00669792	0.008707301	3	0.106614146	0.0877428	9507	ms12008.Bactrocera_kirki
Bactrocera spnMalaysia07	0.00669792	0.006697924	1	0.109766246	0.075737265	6340	ms07797.Bactrocera_nigrita
Bactrocera spnMalaysia08	N/A	N/A	0	0.09720425	0.052243804	3171	ms04673.Bactrocera_digressa
Bactrocera spnMalaysia09	N/A	N/A	0	0.111692602	0.095174263	3171	ms09108.Bactrocera_limbifera
Bactrocera spnSol08	N/A	N/A	0	0.098952301	0.078418231	3171	ms09083.Bactrocera_hantanae
Bactrocera syzygii	0.0074791	0.017426273	1891	0.10186599	0.084831057	192820	ms08867.Bactrocera_propinqua

Bactrocera tenuifascia	0.00793978	0.014075067	78	0.104559046	0.006702413	41067	ms04618.Bactrocera_mayi
Bactrocera thailandica	0.00142938	0.002680965	105	0.062647752	0.022103148	47355	ms03763.Bactrocera_lateritaenia
Bactrocera trilineola	0.00640024	0.012056263	36	0.086642323	0.005358339	28467	ms11141.Bactrocera_caledoniensis
Bactrocera tryoni	0.01205067	0.02545211	325	0.088835405	0	81796	ms07722.Bactrocera_aquilonis
Bactrocera tsatsiae sp n	0.00201072	0.002010724	1	0.126648561	0.107908847	6340	ms10786.Bactrocera_hantanae
Bactrocera tsuneonis	0.00267917	0.002679169	1	0.173966838	0.099799062	6340	ms12078.Bactrocera_minax
Bactrocera tuberculata	0.00685414	0.016075017	378	0.083912771	0.05961152	88032	ms07143.Bactrocera_OTU5353
Bactrocera umbrosa	0.01324608	0.024128686	351	0.124405963	0.10731053	84915	ms07300.Bactrocera_ceylanica
Bactrocera unifasciata	0.00442323	0.00536193	10	0.091869318	0.057602143	15835	ms04491.Bactrocera_manskii
Bactrocera unimacula	0.00809577	0.016104294	28	0.110120332	0.087073007	25312	ms01800.Bactrocera_amarambalensis
Bactrocera usitata	0.01519394	0.030160858	190	0.093193251	0.029490617	63040	ms04491.Bactrocera_manskii
Bactrocera vargasi sp n	0.00938338	0.012064343	3	0.101742218	0.072386059	9507	ms03455.Bactrocera_quasiinfulata
Bactrocera visenda	0.00267917	0.002679169	1	0.144139472	0.131949096	6340	ms04605.Bactrocera_melanothoracica
Bactrocera wuzhishana	0.003441	0.008713137	153	0.107610702	0.088412592	56772	ms12071.Bactrocera_frauenfeldi
Bactrocera xanthodes	0.00089306	0.001339585	3	0.155801406	0.113864702	9507	ms12005.Bactrocera_neoxanthodes
Bactrocera zonata	0.01242535	0.02545211	21528	0.090529688	0.049564635	616512	ms04523.Bactrocera_spMyanmar01
