# Supplementary material for: Novel lures and COI sequences reveal cryptic new species of Bactrocera fruit flies in the Solomon Islands (Diptera, Tephritidae, Dacini)
Source: Zookeys. 2021 Aug 27;1057:49–103. doi: 10.3897/zookeys.1057.68375 (PMC8417025; doi:10.3897/zookeys.1057.68375)
Supplement: Supplementary material 3 — Figure S2. Number of dacine fruit fly species in relation to island size in the Solomon Islands [file zookeys-1057-049-s003.pdf]

Supplementary Figure S-2: Number of Dacine fruit fly species in relation to island size in the Solomon Islands.

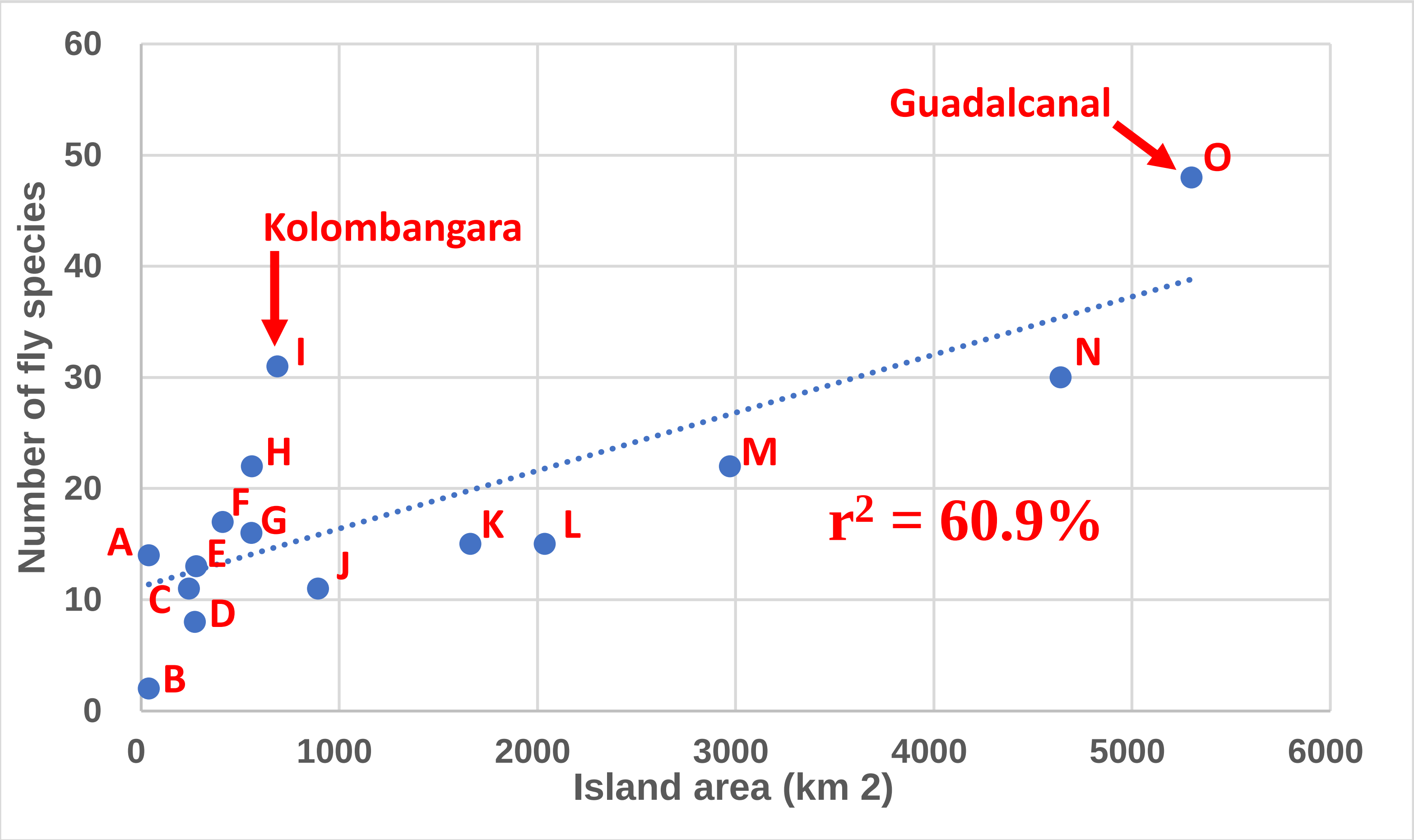

	Island(s)	Area (km^2)	Number of species
A	Gizo	39	14
B	Reef Islands	40	2
C	Vella Lavella	243	11
D	Rennell & Bellona	272	8
E	Russell	280	13
F	Shortland Group	414	17
G	San Cristobal	558	16
H	Florida (Ngella & Savo)	560	22
I	Kolombangara	688	31
J	Santa Cruz	895	11
K	Malaita	1663	15
L	New Georgia	2037	15
M	Choiseul	2971	22
N	Isabel	4640	30
O	Guadalcanal	5302	48
